# Geographic authentication of *Amomum tsaoko* seeds using fourier transform-near infrared spectroscopy combined with machine learning techniques and feature reduction analysis

**DOI:** 10.3389/fpls.2025.1717851

**Published:** 2026-01-22

**Authors:** Yinggang Zheng, Songping Lan, Haoran Hu, Xinwei Huang, Huan Wang, He Cao, Xiaoli Liu, Yaowen Yang, Shengguo Ji, Hui Xie

**Affiliations:** 1 Shanghai Key Laboratory of Anesthesiology and Brain Functional Modulation, Clinical Research Center for Anesthesiology and Perioperative Medicine, Translational Research Institute of Brain and Brain-Like Intelligence, Shanghai Fourth People’s Hospital, School of Medicine, Tongji University, Shanghai, China; 2 School of Traditional Chinese Medicine, Guangdong Pharmaceutical University, Guangzhou, China; 3 State Key Laboratory of Genetic Engineering, School of Life Sciences, Zhangjiang Fudan International Innovation Center, Human Phenome Institute, Fudan University, Shanghai, China; 4 College of Chinese Materia Medica and Yunnan Key Laboratory of Southern Medicinal Utilization, Yunnan University of Chinese Medicine, Kunming, China

**Keywords:** *Amomum tsaoko*, feature reduction analysis, FT-NIR, geographical authentication, machine learning

## Abstract

**Background:**

The dried ripe fruit or seed of *Amomun tsaoko* is a widely used spice and food additive in Eastern and Southeastern Asia. Approximately 90% of the global production of this spice occurs in Yunnan province, China. Over years of cultivation, genetic variations have emerged, leading to wide regional varieties. Authenticating geographical origin has become essential for quality assessment and control, as it directly influences a product’s commercial value.

**Objective:**

This study aims to authenticate the geographical origins of *A. tsaoko* seeds sourced from distinct and narrow geographical regions.

**Methods:**

Near-infrared spectroscopy (NIRS) combined with machine learning (ML) techniques was used to determine the specific geographical origins of *A. tsaoko* seeds.

**Results:**

The results demonstrated that Fourier transform Near-infrared spectroscopy (FT-NIR) followed by a multi-layer perceptron (MLP) was the optimal strategy among all methods tested. This approach achieved a high accuracy of 96.97%. Additionally, feature dimensionality reduction analysis was applied using the Catboost model. This analysis identified certain spectral ranges that contained important features for the model.

**Conclusion:**

This study indicates that pretreatment of NIRS raw data and the use of ML are potential strategies for rapid and specific geographic authentication of plants.

## 1 Introduction

*Amomum tsaoko* Crevost et Lemaire is a perennial herb belonging to the Zingiberaceae family. Its dried ripe fruits and seeds, known as black cardamom or Caoguo, are popular food additives and spices in Eastern and Southeastern Asia. They are widely used specifically in China, Korea, Japan, and Indonesia. In traditional Chinese medicine, these plant parts have been traditionally utilized to treat several ailments. Specific indications include cold-dampness obstruction, epigastric pain and abdominal distension, stuffiness and fullness, vomiting, malaria with cold and fever, and febrile pestilence (The State Pharmacopoeia Commission, 2015; Zhang et al., 2023; Li et al., 2025). Recent pharmacological studies revealed a spectrum of bioactivities for *A. tsaoko*. These include anti-inflammatory properties against insects, antitumor effects in liver cancer cells, anti-angiogenesis effects in ovarian cancer, and constipation-relieving properties (Yang et al., 2008; Kim et al., 2019; Chen et al., 2020; Hu et al., 2023; Ying, 2023). These properties underscore the importance of *A. tsaoko* as a valuable crop serving both culinary and medicinal purposes.

However, increasing demand, coupled with declining wild populations, has heightened concerns regarding the authenticity and geographical origin of *A. tsaoko*. Approximately 90% of *A. tsaoko* is produced in Yunnan province, China (Ma et al., 2017). In this region, variations in climate, topography, and ecology significantly influence fruit morphology, essential oil composition, and concentration (Cui et al., 2017; Wei et al., 2019; Li et al., 2021a). Regional variations in *A. tsaoko* pose a considerable challenge for maintaining the herb’s quality and consistency. Consequently, authenticating the geographical origin of *A. tsaoko* is essential for ensuring quality control and providing consumers with genuine products.

Previous studies have explored methods for authenticating *A. tsaoko*. For example, gas chromatography-mass spectrometry (GC–MS) (Qin et al., 2021) and molecular marker techniques, including simple sequence repeats (SSR) and expressed sequence tag-simple sequence repeats (EST-SSR) (Ma et al., 2022), have been applied. However, current studies have primarily focused on distinguishing between different species, such as *A. tsaoko*, *A. paratsao-ko*, and other Zingiberaceae family plants, instead of authenticating their specific geographical origins. Furthermore, typical methods for geographical tracing typically operate at a coarse resolution (for example, discriminating between provinces or cities). They lack the precision needed to differentiate among more localized growing regions (Liu et al., 2021a). In previous studies, our group developed a multi-element fingerprinting method by utilizing absolute quantification of elements in *A. tsaoko* seeds, which enabled the geographical authentication of *A. tsaoko* seed samples (Liu et al., 2023). While this approach demonstrated strong performance, it faced several limitations, such as high cost, complex sample preparation, reliance on specialized instrumentation, low throughput, and potential sample destruction. To clearly contextualize the novelty and advancement of the current study, a comparative summary of prior research on *A. tsaoko* authentication is provided in **Supplementary Table 1**. These challenges underscore the need for a more efficient and accessible method for geographical authentication.

NIRS was established in the 1960s for cereal analysis (Agelet et al., 2010). Currently, NIRS is widely used for the authentication of geographical origin (Liu et al., 2021b; Nguyen Minh et al., 2022; Schütz et al., 2022; Santos-Rivera et al., 2024). For instance, it has been successfully used to distinguish varieties of millet sourced from different regions of China (Kabir et al., 2021). It offers several advantages such as low cost, rapid operation, high throughput, and non-destructive testing. These advantages make it an ideal solution for quality control and geographical authentication of *A. tsaoko* seeds. To date, NIRS has been effectively utilized to identify the drying temperatures of *A. tsaoko* (He et al., 2023).

In this study, we propose a protocol that integrates Fourier Transform Near-infrared spectroscopy (FT-NIR) with machine learning techniques to precisely identify and trace the narrow geographical origin of *A. tsaoko* seeds. Our primary objective was to establish an accurate and interpretable model for geographical authentication. To achieve this, we leveraged explainable artificial intelligence for optimal feature selection and conducted a comprehensive comparison of multiple machine learning algorithms to identify the most effective classifier.

## 2 Materials and methods

### 2.1 Samples information

**Figure 1:**
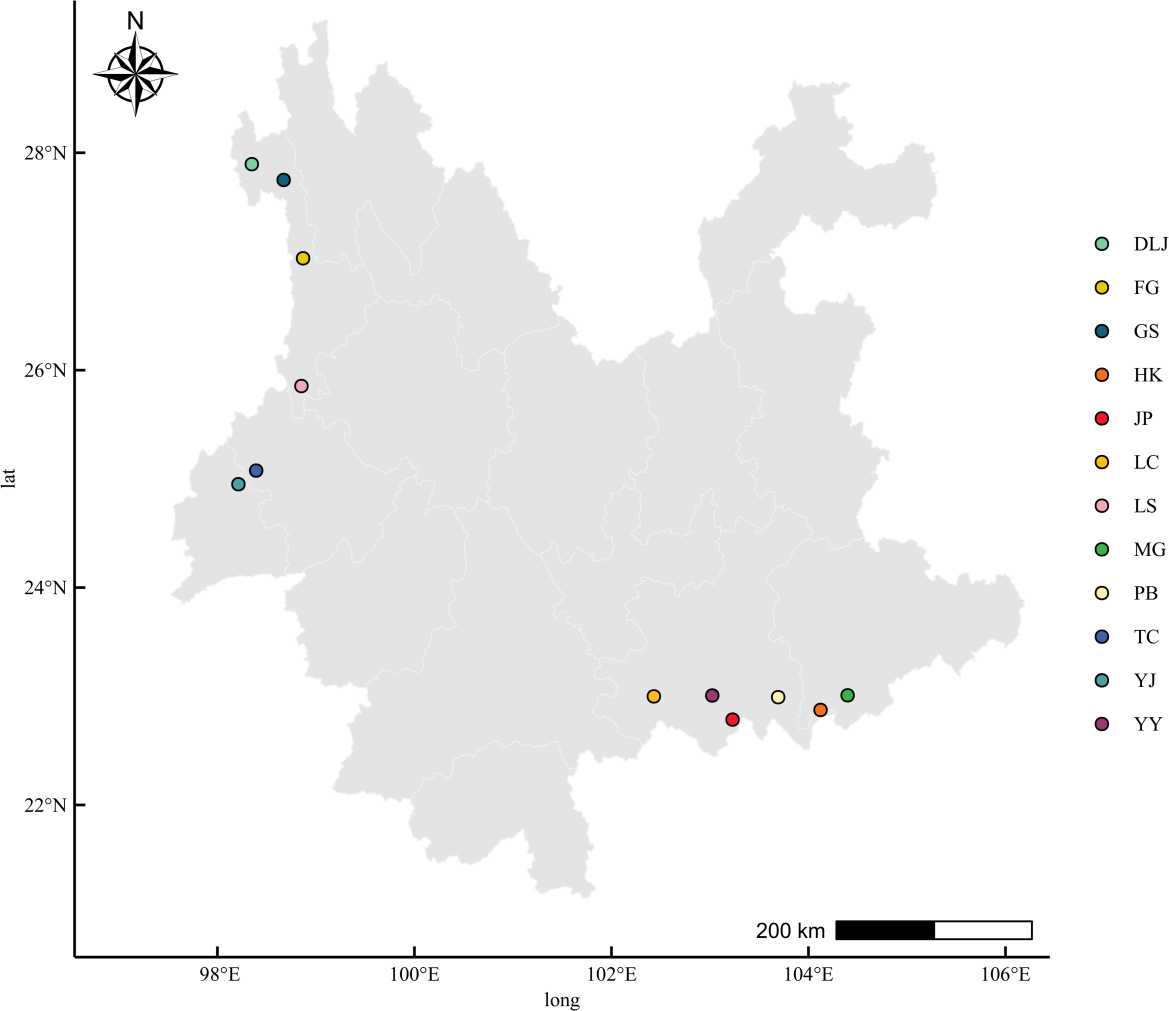
Geographical location information of the collected *A. tsaoko* fruits.

The plant material used in this study was the same as previously reported (Liu et al., 2023). Briefly, in autumn 2018, *A. tsaoko* fruits were collected from 12 populations. The botanical identity was confirmed by Professor Yaowen Yang. The geographical origin for each sample was not determined by expert opinion but was an objective, ground-truthed fact based on its precise GPS-recorded collection location (**Figure 1**). This verifiable geographical label served as the definitive benchmark for our classification model. The geographical separation among these populations was relatively small. For example, the shortest inter-population distance was 22.9 km, occurring between Tengchong (TC) and Yingjiang (YJ) sites (**Figure 1**). The voucher specimens were deposited at the Yunnan University of Chinese Medicine Museum. The morphological characteristics of the individual fruit and powdered seed samples from all 12 geographical origins are visually documented in **Supplementary Figure 1**. Three fruit pools were created from six or more randomly selected plants in each population. The 36 fruit samples were then dried until their mass stabilized. The seeds were extracted from the fruits, then ground and sifted. The resulting powder materials (50–65 mesh) were stored in sealed sample bottles at 4 °C pending subsequent analysis. Each individual pooled powder sample was subjected to spectral acquisition in triplicate (three technical replicates), resulting in a total dataset of 108 spectra (12 populations × 3 pooled biological replicates × 3 technical replicates).

### 2.2 FT-NIR spectra acquisition and data pretreatment

FT-NIR spectra were obtained using a Bruker TANGGO FT-NIR spectrometer (Bruker, Karlsruhe, Germany) equipped with a diffuse integrating sphere and a sample rotator. The scan ranged from 12,000 to 4,000 cm^–1^ at a resolution of 8 cm^–1^. Samples were meticulously placed in quartz cups, filling approximately two-thirds of each cup’s volume. Before collecting the sample spectra, the spectral information of the background was collected in air to exclude H_2_O and CO_2_ interference. Each sample underwent 64 scans at 22 °C with a relative humidity of 25%–30%. All experiments were conducted in triplicate, resulting in a total of 108 samples, which were used as the dataset for this study. Data preprocessing was subsequently conducted using Omnic and TQ software (Thermo Fisher Scientific, MA, USA) prior to model development and validation. The final dataset for machine learning consisted of 108 samples (rows), with each sample characterized by 1900 spectral data points (wavenumbers) acting as features (columns).

### 2.3 Feature selection

A feature screening was performed using six ML algorithms combined with SHAP: CatBoost (Dorogush et al., 2018), Decision Tree (Utgoff, 1989) (DT), Extra Trees (Sharaff and Gupta, 2019) (ET), Light Gradient Boosting Machine (Fan et al., 2019) (LightGBM), Random Forest (Tin Kam, 1995) (RF), and eXtreme Gradient Boosting (Chen and He, 2015) (XGBoost). For each algorithm, the top 20 features ranked by SHAP importance were initially selected. Subsequently, the ten most important features from this subset were retained. This multi-step screening process yielded a final set of ten spectral features. These features were subsequently used for model construction.

### 2.4 Model development and validation

All ML experiments were conducted on a Windows 10 computer using an algorithm implementation framework based on Python (version 3.10) and R (version 4.3.3). To ensure a robust and comprehensive benchmark, a total of 12 classification algorithms were evaluated. This set encompassed a wide range of machine learning paradigms, including Logistic Regression (LaValley, 2008) (LR), CatBoost, DT, RF, AdaBoost (Freund and Schapire, 1997), ET, Support Vector Machine (Patle and Chouhan, 2013) (SVM), Gaussian Naive Bayes (Webb et al., 2010) (NB), K-Nearest Neighbors (Liao and Vemuri, 2002) (KNN), Multi-layer Perceptron (Yang, 2010) (MLP), XGBoost, and LightGBM. This approach allowed for a systematic comparison to identify the most performant model for our specific dataset without *a priori* bias. The key Python packages utilized in this study, along with their versions, included: scikit-learn (1.6.1) for traditional machine learning models and hyperparameter tuning; TensorFlow (2.8.1) and PyTorch (2.6.0+cu118) for deep learning model development (CNN and Transformer); CatBoost (1.2.7), LightGBM (4.6.0), and XGBoost (2.1.4) for gradient boosting algorithms; and SHAP (0.47.2) for feature importance analysis, with additional support from pandas (2.2.3) and NumPy (1.24.0) for data manipulation. To ensure full reproducibility, the complete code and a detailed environment configuration file have been deposited in the GitHub repository (https://github.com/aibiobrain/FT-NIR).

Given the constraints of a limited sample size, which is common in spectroscopic studies, we employed a rigorous validation strategy to thoroughly assess model stability and generalizability. During the experiments, training and validation were conducted under both cross-validation and hold-out methods. We varied the training set and test set ratios (8:2, 7:3, and 6:4) to examine the sensitivity of model performance to different data partitioning scenarios. This practice helps ensure that the reported performance is not an artifact of a single, fortunate data split. To further verify statistical robustness, we selected 10 different random seeds for repeated experiments with each splitting ratio. Hyperparameter tuning was conducted for all models using the RandomGridSearch algorithm from the scikit-learn library.

We also compared two deep learning models, a Convolutional Neural Network (CNN) and a Transformer network, using a categorical cross-entropy loss function and accuracy as the evaluation metric. The detailed architectures of these models are provided in **Supplementary Figure 2**, **3**. In the CNN architecture, the input 10-dimensional feature vector was processed through three sequential 1D convolution layers with channel numbers optimized over (32, 64, 128) (64, 128, 256), and (128, 256, 512). A maximum pooling layer was used for dimensionality reduction, with batch normalization and dropout layers incorporated to prevent overfitting. The classification was performed by a fully connected output layer with 12 neurons and a Softmax activation function. In the Transformer architecture, the input features were first converted into a 64-, 128-, or 256-dimensional embedding. Feature modeling was then performed through 2, 3, or 4 Transformer blocks, each employing a multi-head attention mechanism (2, 4, or 8 heads) to capture feature correlations. Each attention layer was followed by a feedforward network, with layer normalization and residual connections to stabilize training. Finally, global average pooling was applied before the classification layer to output the results.

### 2.5 Model evaluation

To comprehensively assess the model’s predictive ability, a set of core evaluation metrics is adopted. The evaluation metrics include accuracy, precision, recall, F1-score, and the area under the receiver operating characteristic curve (AUC). These metrics focus on different aspects of model performance, and their combined use prevents one-sided judgments caused by relying on a single metric.

### 2.6 External validation and robustness assessment

To further assess the model’s generalization ability and address concerns regarding the limited sample size, we performed external validation using data augmentation techniques. Given the high-dimensional nature of the raw spectral data (>100 dimensions) and the small number of samples per class, direct application of oversampling algorithms could be unreliable. Therefore, we first applied our established feature selection procedure (Section 2.3) to reduce the dimensionality to the top 10 most important features. To preliminarily assess the model’s generalizability beyond the single-season dataset and mitigate the impact of limited sample size, we employed the SMOTE for external validation. The external validation procedure was as follows: 1. Model Training: The optimal Multi-Layer Perceptron (MLP) model was first trained on the entire original dataset (108 samples) using the previously identified top 10 features and the best-performing hyperparameters. 2. Synthetic Dataset Generation: Using the imbalanced-learn library (v0.10.1) in Python, we synthetically oversampled this external set to create a balanced validation dataset with 18 samples per class, resulting in a total of 216 synthetic samples across 12 classes. This process allowed us to simulate a scenario with a larger, unseen validation set. 3. Generalization Assessment: The pre-trained MLP model (from Step 1) was then used to predict the geographical origins of the synthetically generated samples in the augmented external validation set. For comparison, we also evaluated two alternative resampling methods: Random Oversampling (simple duplication) and Bootstrapping (sampling with replacement). The performance of our optimal classifier (MLP) was then evaluated on these augmented datasets to ensure robustness and to verify that the synthetic samples maintained the statistical characteristics of the original data.

### 2.7 GC-MS analysis of the essential oil

It was reported in our previous paper (Li et al., 2021a). The GC-MS analysis of the essential oil was carried out using an Agilent 9000 GC system coupled with an Agilent 5977B MSD. The sample was introduced into the instrument through split injection with a pressure of 25 psi. at 280 °C, and the injection volume was 1 μL. Compounds were separated along a HP-5MS capillary column (30 m × 0.25 mm × 0.25 μm film thickness). GC-MS data was obtained from split ratios of 44:1 and 8:1. The carrier gas was helium at a flow rate of 1.0 mL/min. MS detection was obtained in an electron impact mode at 70 eV. The temperature of the MS transfer line, quadrupole, and ion source were set at 280 °C, 150 °C, and 230 °C, respectively. The full scan *m*/*z* range was 15–300 Da. In the sequence, the samples were run randomly, and one QC injection was added after every seven sample injections to ensure data stability.

## 3 Results and discussion

### 3.1 FT-NIR spectra

**Figure 2:**
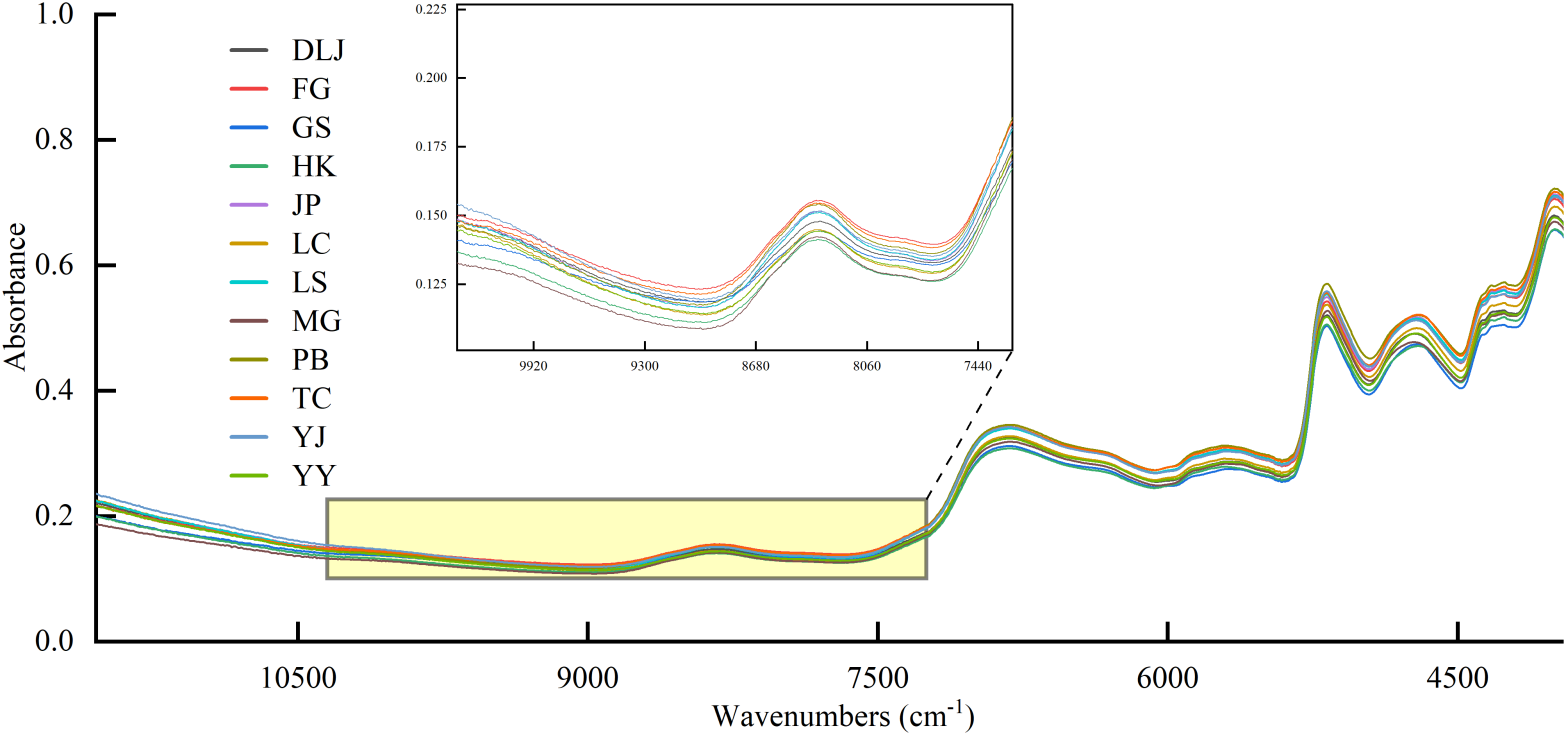
The raw spectra of *A. tsaoko* seeds.

The raw spectra of *A. tsaoko* seeds were derived from twelve populations (**Figure 2**). Despite the substantial overlap and similarity in their raw spectral data, the samples exhibited high-dimensional absorbance features. These feature vectors enabled the classification of the geographic origins of the twelve samples. Differences in peak values were observed across regions (**Figure 2**). Notably, even within the wavenumber range of 7740 to 9920 cm^-1^, where spectral variations are subtle and not easily distinguished by visual assessment, consistent variations are still present. However, these subtle, consistent spectral differences enable the use of ML to distinguish the regional origins of *A. tsaoko* seeds. The high-dimensional absorbance data provide a sufficient number of informative features to facilitate classification among the twelve geographical origins.

### 3.2 Feature selection and model performance

**Figure 3:**
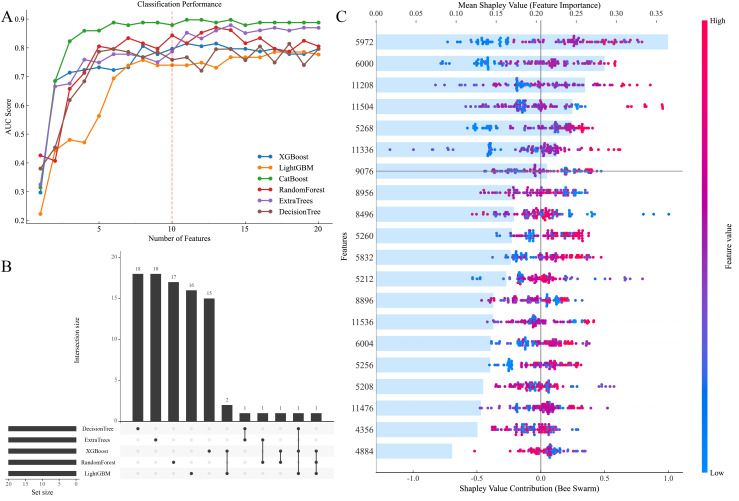
**(A)** Characteristic filtering of different methods, **(B)** upset plot of overlap features of different methods, **(C)** SHAP values of the optimal model Catboost with feature importance plots for the top 20 seed profiles.

Model accuracy improved rapidly with the inclusion of top-ranked features. However, it plateaued after approximately 10 features, indicating that additional inputs provided minimal improvement (**Figure 3A**). We also compared the top 20 most important features across the six ML models and observed notable differences in the characteristic wavelengths prioritized by each algorithm (**Figure 3B**). Based on AUC performance, the CatBoost was selected for feature filtering. The top 10 features identified by the model were subsequently used in the analysis (**Figure 3C**). Taking the most important feature at 5972 cm^–1^, as an example, the sample exhibits a lower peak revealed in blue and a higher peak in red. These peaks are separated on opposite sides of the horizontal line, indicating that their intensities differ between sample types. This distinction allows for effective classification of the samples.

**Figure 4:**
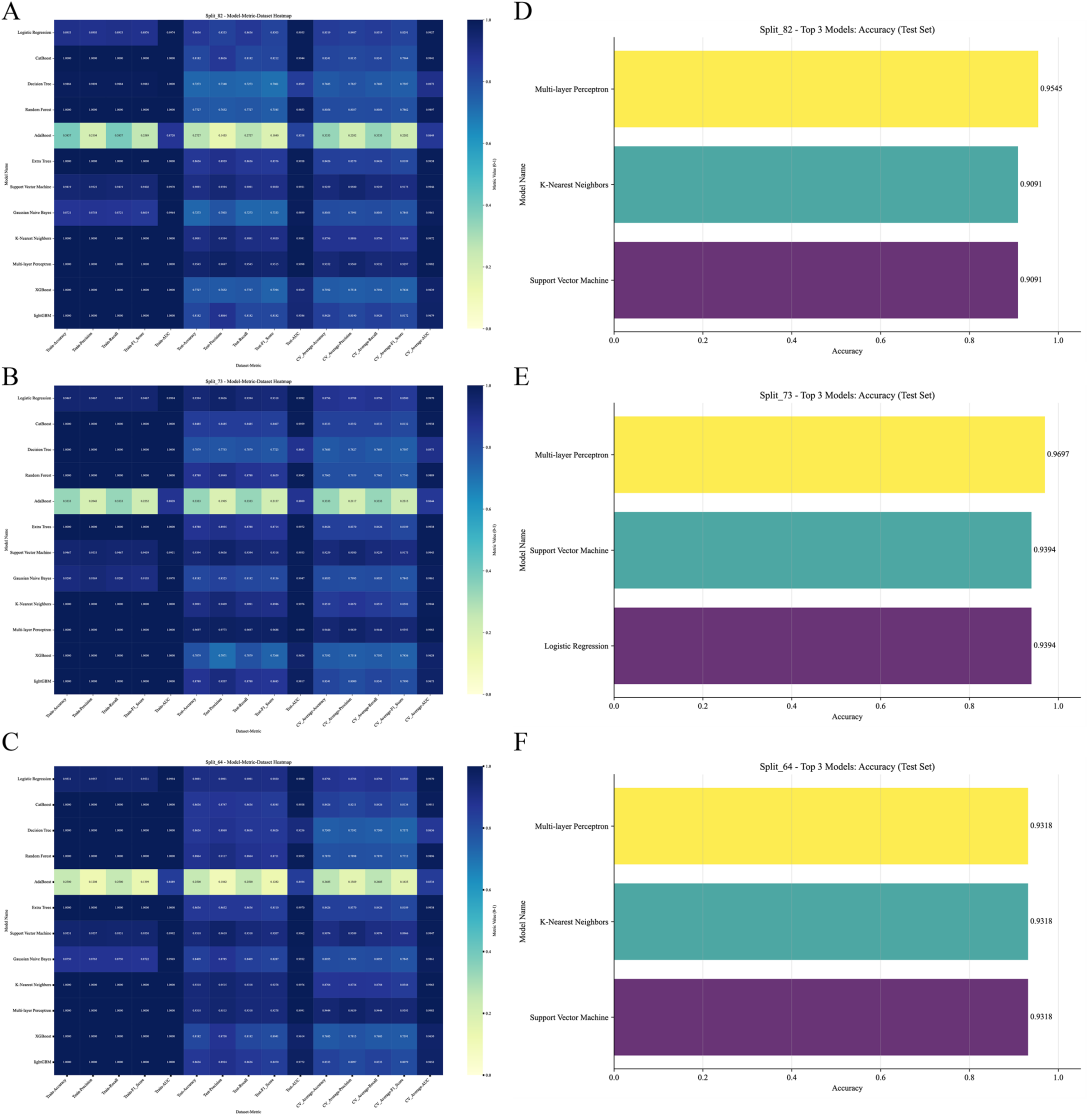
Comparative performance evaluation of 12 machine learning algorithms under different dataset splitting schemes. **(A–C)** Heatmaps of core evaluation metrics (including AUC, accuracy, precision, recall, and F1-score) for all models under **(A)** 8:2, **(B)** 7:3, and **(C)** 6:4 training-to-test set split ratios. **(D–F)** Bar plots showing the prediction accuracy of the top three performing models under the **(D)** 8:2, **(E)** 7:3, and **(F)** 6:4 split ratios, respectively.

To evaluate the performance of different machine learning models in classifying the geographical origins of *A. tsaoko* seeds, we used the Randomized Grid Search algorithm from scikit-learn for hyperparameter optimization. A total of 12 ML models were trained and evaluated using a comprehensive set of performance metrics. The models were evaluated under both 3-fold cross-validation and hold-out validation schemes, with training-to-test set ratios of 8:2, 7:3, and 6:4. The resulting metrics, including accuracy, precision, recall, and F1-score for the training set, validation set, and cross-validation average, are summarized in a heatmap (**Figures 4A–C**). The top three models with the highest accuracy across the different data splits are further visualized (**Figures 4D–F**). Among all configurations, the MLP model achieved the highest identification accuracy of 96.97% under the 7:3 split ratio (**Figure 4F**), and was selected as the optimal algorithm for this geographical traceability task.

**Figure 5:**
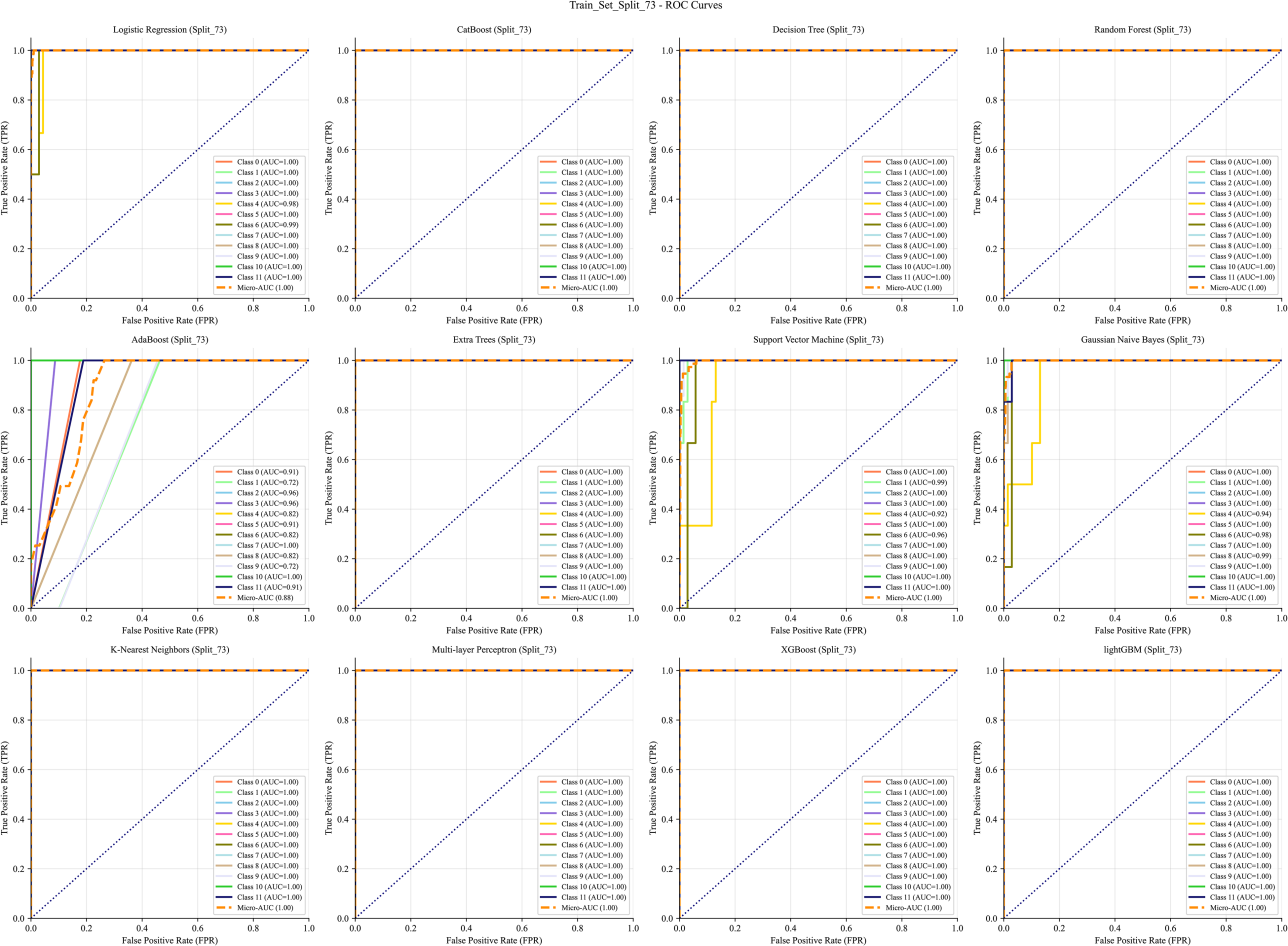
The ROC curve of the training set with 12 machine learning algorithms. The algorithms, in order, are: Logistic Regression (LR), CatBoost, Decision Tree (DT), Random Forest (RF), AdaBoost, Extra Trees (ET), Support Vector Machine (SVM), Gaussian Naive Bayes (NB), K-Nearest Neighbors (KNN), Multi-layer Perceptron (MLP), XGBoost, and LightGBM.

**Figure 6:**
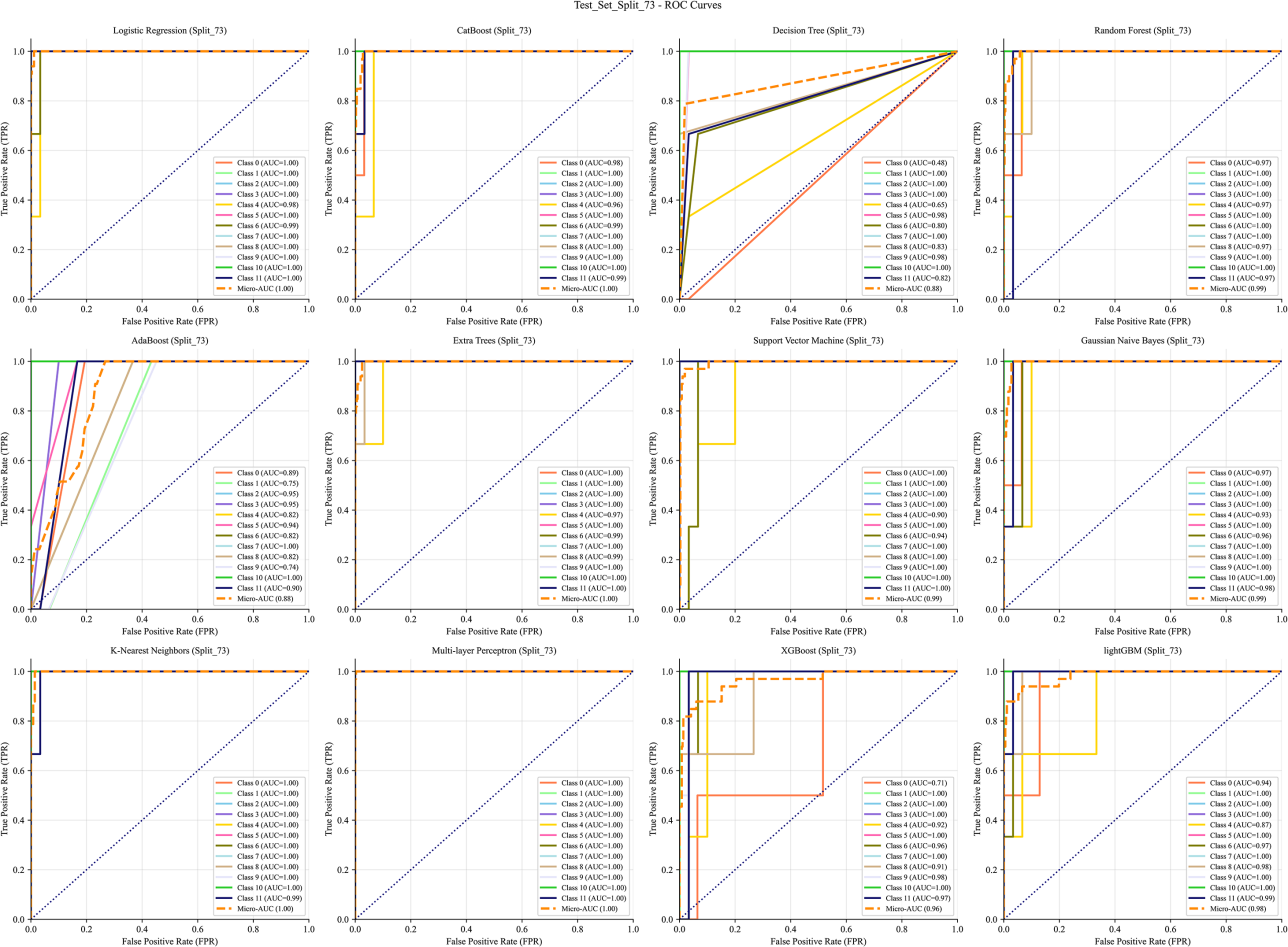
The ROC curve of the test set with 12 machine learning algorithms. The algorithms, in order, are: Logistic Regression (LR), CatBoost, Decision Tree (DT), Random Forest (RF), AdaBoost, Extra Trees (ET), Support Vector Machine (SVM), Gaussian Naive Bayes (NB), K-Nearest Neighbors (KNN), Multi-layer Perceptron (MLP), XGBoost, and LightGBM.

**Figure 7:**
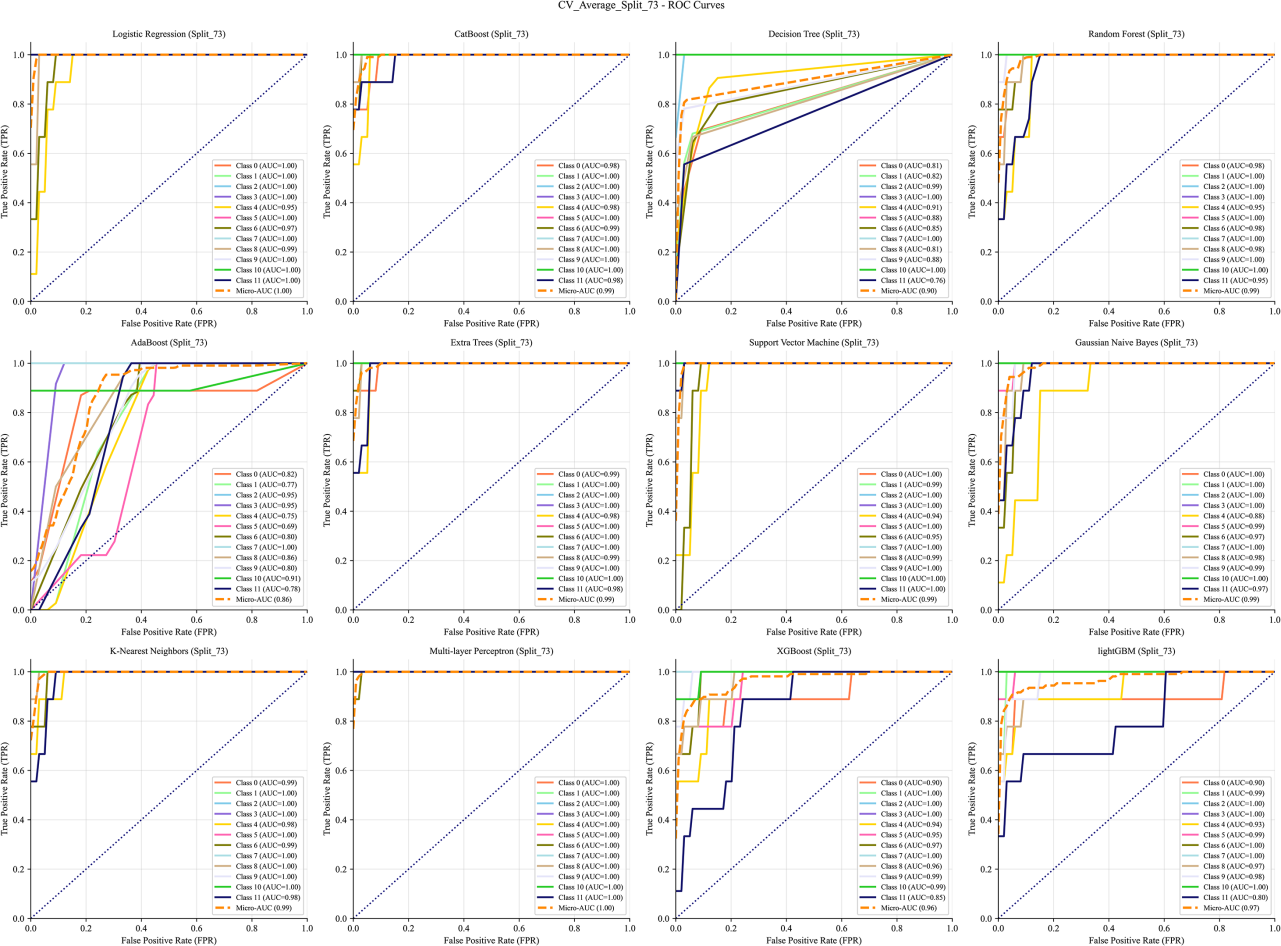
The ROC curve of the cross-validation with 12 machine learning algorithms. The algorithms, in order, are: Logistic Regression (LR), CatBoost, Decision Tree (DT), Random Forest (RF), AdaBoost, Extra Trees (ET), Support Vector Machine (SVM), Gaussian Naive Bayes (NB), K-Nearest Neighbors (KNN), Multi-layer Perceptron (MLP), XGBoost, and LightGBM.

**Figure 8:**
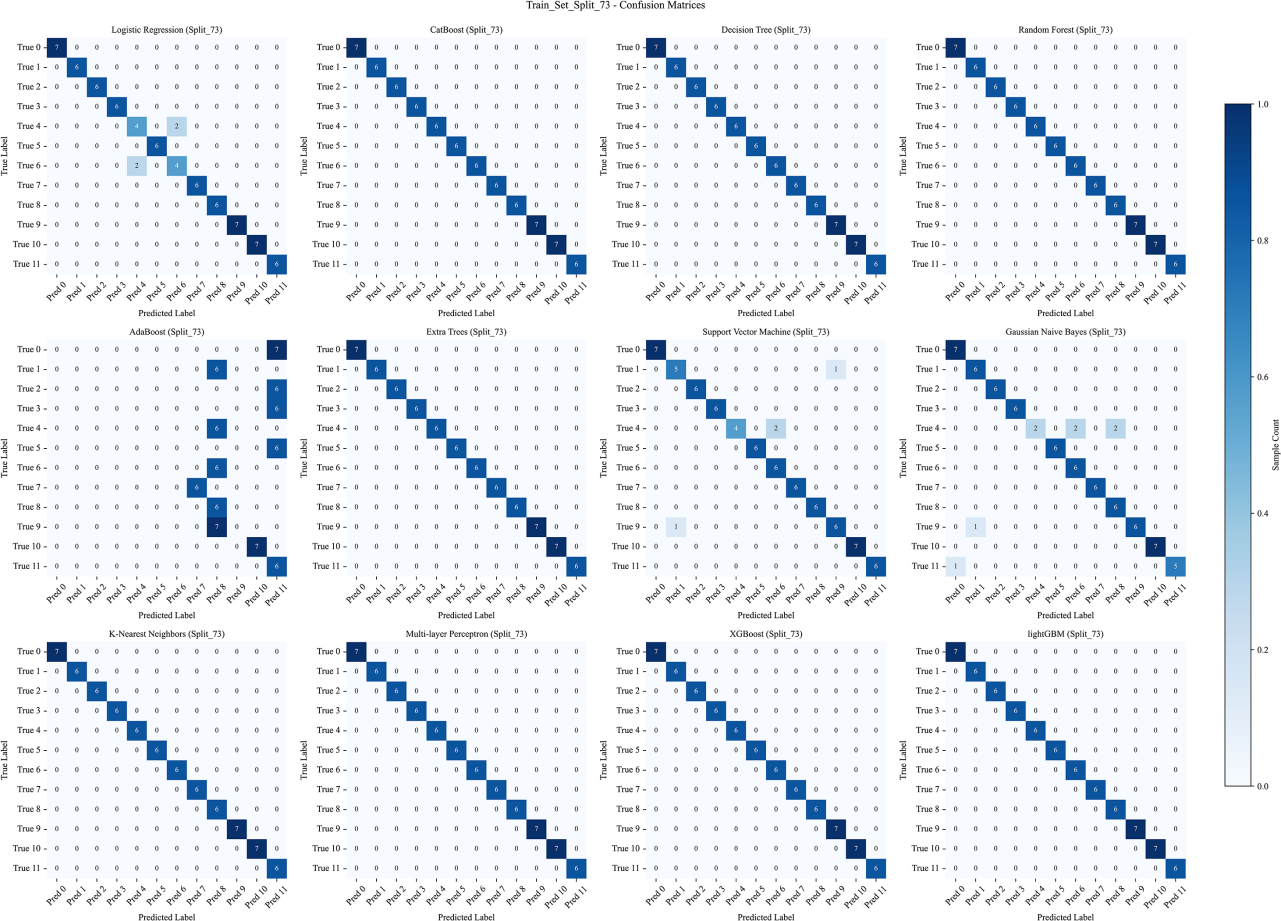
The confusion matrix of the training set with 12 machine learning algorithms. The algorithms, in order, are: Logistic Regression (LR), CatBoost, Decision Tree (DT), Random Forest (RF), AdaBoost, Extra Trees (ET), Support Vector Machine (SVM), Gaussian Naive Bayes (NB), K-Nearest Neighbors (KNN), Multi-layer Perceptron (MLP), XGBoost, and LightGBM.

**Figure 9:**
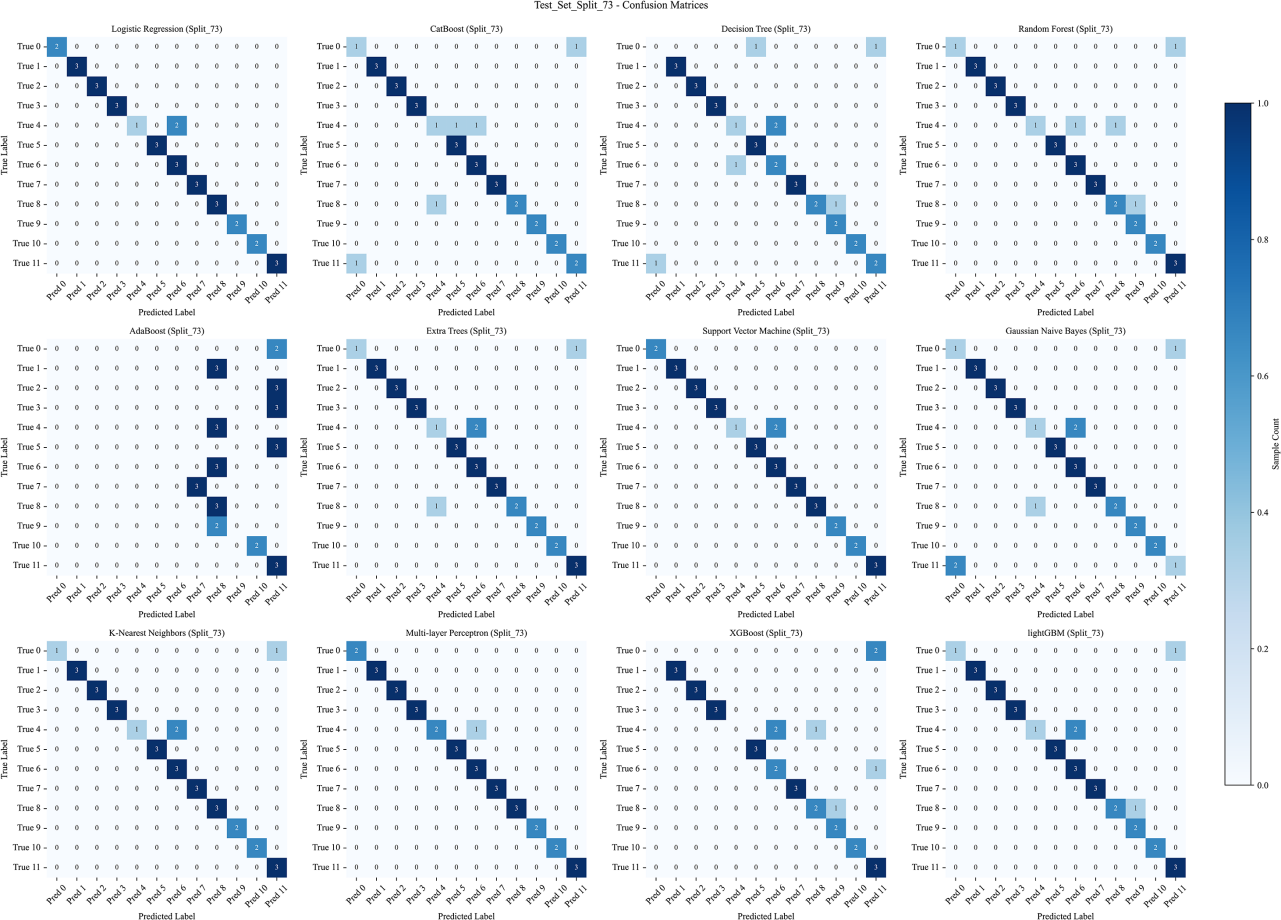
The confusion matrix of the test set with 12 machine learning algorithms. The algorithms, in order, are: Logistic Regression (LR), CatBoost, Decision Tree (DT), Random Forest (RF), AdaBoost, Extra Trees (ET), Support Vector Machine (SVM), Gaussian Naive Bayes (NB), K-Nearest Neighbors (KNN), Multi-layer Perceptron (MLP), XGBoost, and LightGBM.

**Figure 10:**
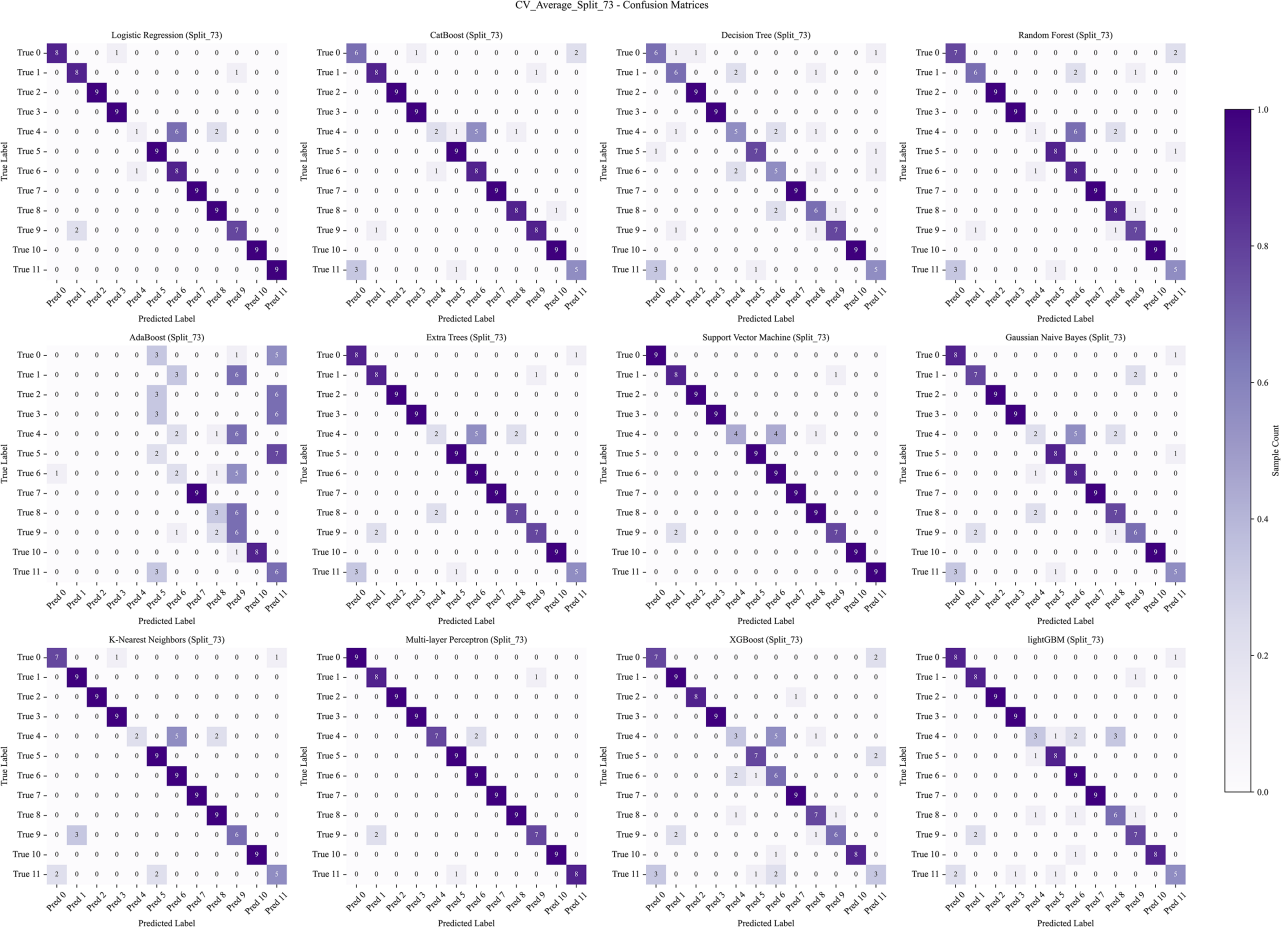
The confusion matrix of the cross-validation with 12 machine learning algorithms. The algorithms, in order, are: Logistic Regression (LR), CatBoost, Decision Tree (DT), Random Forest (RF), AdaBoost, Extra Trees (ET), Support Vector Machine (SVM), Gaussian Naive Bayes (NB), K-Nearest Neighbors (KNN), Multi-layer Perceptron (MLP), XGBoost, and LightGBM.

**Figure 11:**
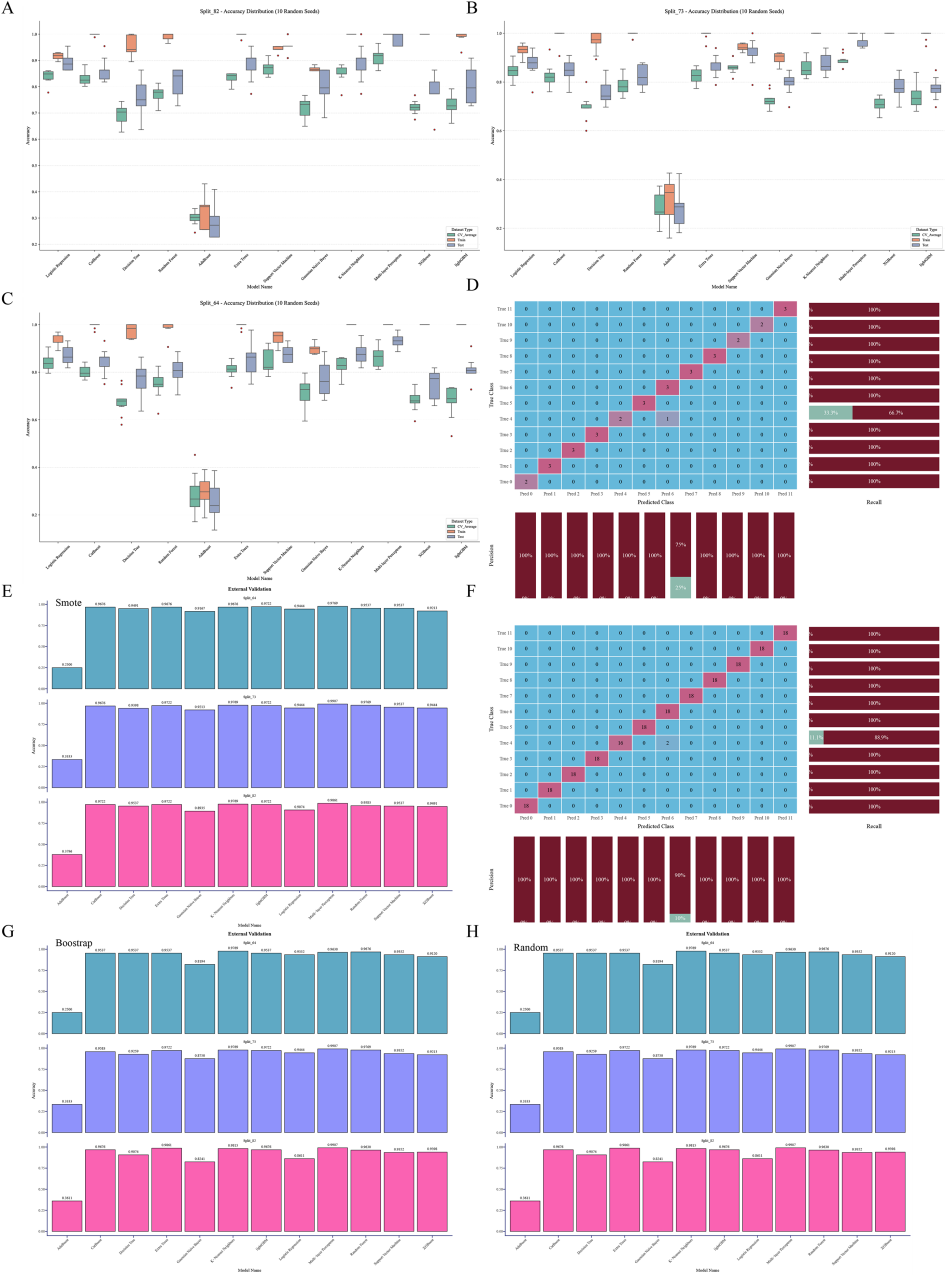
Model robustness assessment and external validation. **(A–C)** Box plots of classification accuracy across 10 independent runs with different random seeds, demonstrating model stability under **(A)** 8:2, **(B)** 7:3, and **(C)** 6:4 training-test split ratios. **(D)** Confusion matrix of the optimal Multi-layer Perceptron (MLP) model, annotated with class-specific precision and recall values. **(E)** Prediction accuracy of the MLP model on the externally augmented validation set generated via the Synthetic Minority Oversampling Technique (SMOTE). **(F)** Confusion matrix illustrating the model’s performance on the SMOTE-augmented external validation set. **(G, H)** Prediction accuracy of the MLP model on the externally augmented validation set generated via the bootstrap oversampling data and random oversampling data.

The data were split into training and test sets using a 7:3 ratio. Receiver operating characteristic (ROC) curves generated from 12 ML algorithms–across training, test, and cross-validation datasets indicated that MLP achieved the best performance (**Figures 5**–**7**). Furthermore, the Confusion Matrix of different classes indicated that MLP is the best algorithm (**Figures 8**–**10**). To assess the robustness and stability of all models, we repeated the training process using 10 different random seeds for each data splitting ratio. The results were summarized using accuracy boxplots (**Figures 11A–C**). Within this assessment, the MLP model exhibited the best and most stable performance under the 7:3 splitting scheme, displaying minimal variance in accuracy across iterations (**Figure 11B**).

**Figure 12:**
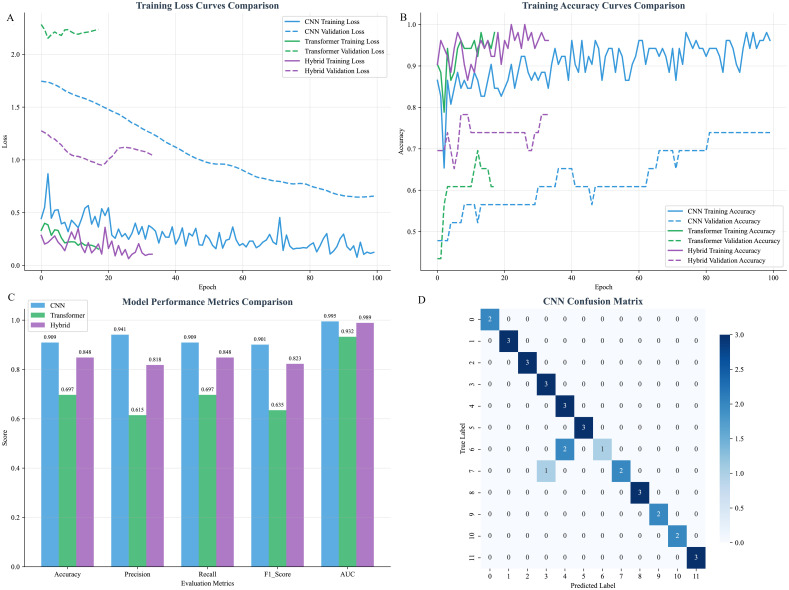
Comparative performance analysis between CNN, Transformer and hybrid models. **(A)** Training loss curves of the CNN, Transformer and hybrid models over 100 epochs. **(B)** Training accuracy curves throughout the training process. **(C)** Quantitative evaluation of model performance on the independent test set. **(D)** Confusion matrix of the CNN model on the test set, detailing the per-class classification results (True Class vs. Predicted Class).

To further explore the potential of automatic feature extraction, we implemented and rigorously compared three deep learning architectures: a CNN, a Transformer, and a hybrid CNN-Transformer model. The training dynamics, as reflected in the loss and accuracy curves (**Figures 12A, B**), clearly demonstrated that the CNN model achieved the most stable convergence and the best performance among the deep learning approaches. On the independent test set, the CNN model substantially outperformed the others, achieving a high identification accuracy of 90.9% (**Figures 12C, D**). In contrast, the Transformer model struggled with this task, attaining an accuracy of only 69.7%. The hybrid CNN-Transformer architecture, designed to leverage both local feature extraction and global contextual modeling, achieved an intermediate accuracy of 84.8%. This result indicates that while integrating Transformer modules provides a benefit over the standalone Transformer, it still falls short of the performance delivered by the simpler, yet highly effective, CNN model for our specific dataset. Ultimately, however, the top-performing MLP model, which achieved 96.97% accuracy, surpassed even the best deep learning model, underscoring the exceptional efficacy of our feature selection strategy combined with traditional machine learning for this specific task.

To promote practical application, the final MLP model was implemented into a simplified prediction tool. This tool integrates the top 10 seed profiles of *A. tsaoko* (5972, 6000, 11208, 11504, 5268, 11336, 9076, 8956, 8496, 5260 cm^–1^). The integration enables rapid, non-invasive prediction of *A. tsaoko* location. The web application of this AI model is accessible online at https://frp-act.com:44782/.

### 3.3 External model validation

To address potential concerns about model generalizability given the single-season dataset, we conducted an external validation exercise using synthetic data augmentation on the optimal 10-feature subset. After dimensionality reduction, SMOTE was applied to generate an expanded validation set. The distributions of the synthetic samples in the reduced feature space were visually and statistically consistent with the original data, confirming that no significant distortion was introduced (**Supplementary Figure 4**-**6**). We compared the model’s performance when validated on datasets augmented via SMOTE, Random Oversampling, and Bootstrapping. The optimal MLP model maintained high and comparable accuracy across all three augmented validation sets (**Figures 11E-H**), with the SMOTE-augmented set yielding an accuracy of 94.4%. This result demonstrates that within the carefully selected, low-dimensional feature space, SMOTE provided a valid mechanism for robustness assessment without compromising data reliability.

### 3.4 Correlation between key spectral features and metabolites

Due to the overlapping nature of bands within each signal, it is not possible to attribute a distinct wavenumber to a single substance in the metabolome (Wang et al., 2020). However, chemical bonds are still sufficient to provide abundant spectral information. The FT-NIR spectra reveal absorption peaks commonly associated with various molecular vibrations (**Table 1**). Putative assignments based on common spectral libraries suggest that the range of 4250~4350 cm^-1^ may correspond to the stretching vibrations of C-H and C-C combinations, which are present in various terpenes. The spectral region of 5000~5200 cm^-1^ is often associated with the first overtones of C=O stretching vibrations, while absorptions between 5300~6000 cm^-1^ are typically attributed to the overtones of C-H and CH_2_ stretching vibrations. The broad feature around 6500~7300 cm^-1^ can be assigned to the first overtone of O-H stretching vibrations. The observed spectral differences across origins suggest underlying variations in chemical composition, thereby supporting the feasibility of FT-NIR-based geographical authentication.

**Table 1:** Organic groups absorbed indifferent frequency ranges.

Wavenumber(cm^-1^)	Vibrating group	Main structure
6500~7300	O-H stretching vibration first-order overtone	Geraniol, Nerolidol and α-Terpineol
6000~5300	Combined frequencies of vibrations such as C-H and CH2	–
5000~5200	C=O, etc.	Citral , (2E)-2-Decenal
4250~4350	C-H, etc.	α-Pinene, β-Pinene, and 1,8-Eudesmolgin

To enhance the biological interpretability of the important wavenumbers identified by the SHAP analysis, we investigated their potential correlations with known metabolites. We used GC-MS to measure the critical volatile esters. Then we performed a correlation analysis between the absorbance values of the top 10 spectral features (5972, 6000, 11208, 11504, 5268, 11336, 9076, 8956, 8496, 5260 cm^-1^) and the concentrations of a series of flavonoids and volatile aroma compounds obtained from our previous metabolomic studies on the same set of *A. tsaoko* samples (Li et al., 2021b; Wang et al., 2021).

**Figure 13:**
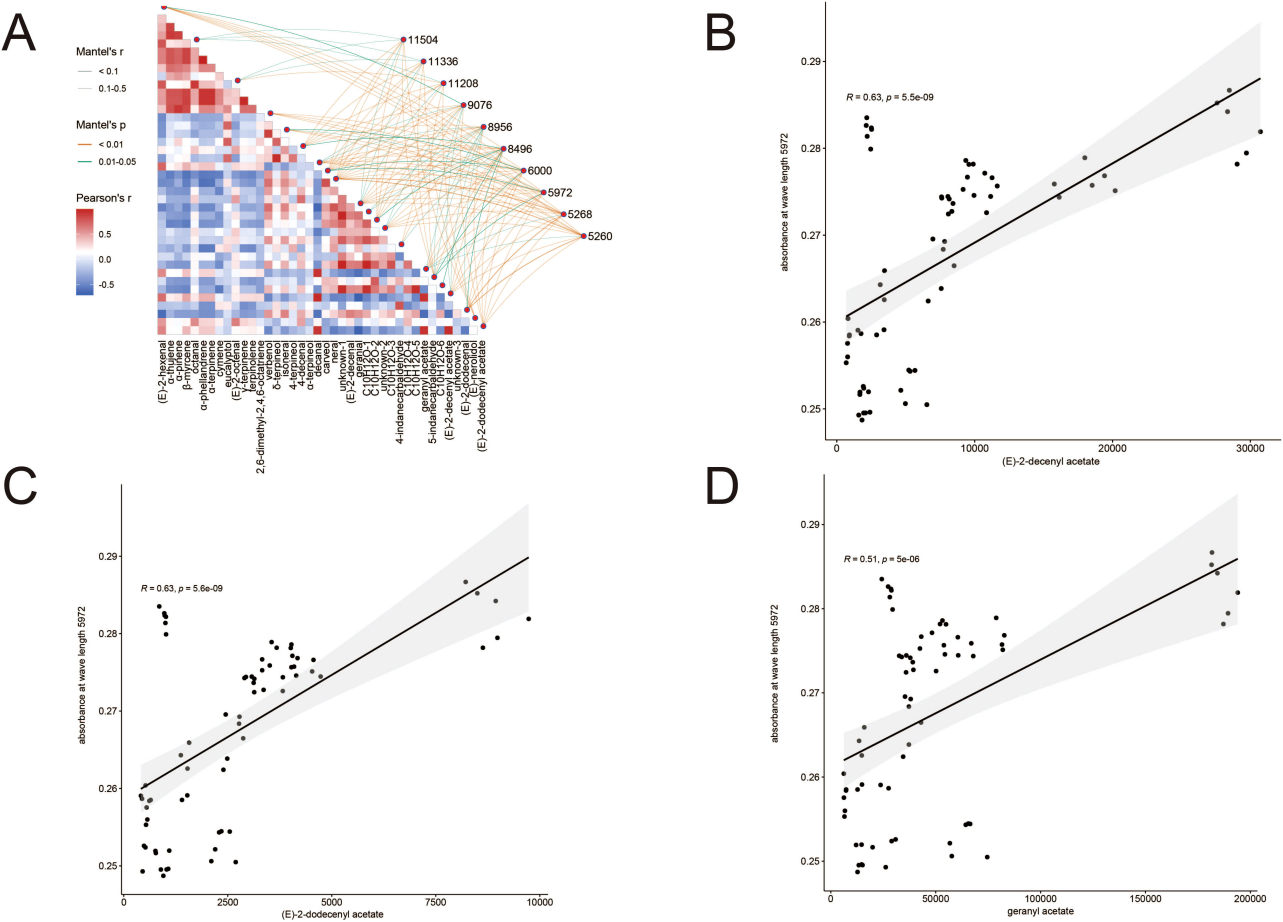
**(A)** Heatmap of Mantle correlation coefficients between the top 10 most important infrared wavelengths and selected flavor metabolites. **(B-D)** Scatter plots with fitted regression lines illustrating the significant correlations between the specific infrared wavelength at 5972 cm^-1^ and three key acetate esters: **(B)** (E)-2-decenyl acetate, **(C)** (E)-2-dodecenyl acetate, and **(D)** geranyl acetate. The strong correlations suggest the potential of using the 5972 cm^-1^ wavelength as a key marker for predicting the concentrations of these flavor compounds.

The results revealed that the key spectral features were broadly correlated with a diverse range of flavonoids (**Figure 13A**). More specifically, the important wavenumber 5972 cm^-1^ showed strong positive correlations with several critical volatile esters, including (E)-2-decenyl acetate, (E)-2-dodecenyl acetate, and geranyl acetate (**Figure 13B-D**). These compounds are well-characterized as key aroma constituents that define the distinctive flavor profile of *A. tsaoko* (Li et al., 2021b; Wang et al., 2021).

This finding provides a plausible biochemical basis for the high discriminatory power of the 5972 cm^-1^ feature. It suggests that our FT-NIR model, guided by machine learning, is effectively capturing intrinsic chemical variations in important flavor-related metabolites, which are themselves influenced by geographical growing conditions. This linkage significantly strengthens the scientific value of our spectral authentication model by connecting spectral patterns to tangible quality attributes.

## 4 Discussion

The quality of plant-derived products often varies across different regions (Dhami and Mishra, 2015; Jangra et al., 2021; Nolden and Forde, 2023; Benomar et al., 2025). Such variation typically stems from a complex interplay of factors, including genetic background, ecological conditions, and human cultivation practices (Wang et al., 2023; Liu et al., 2024; Hu et al., 2025). Due to these inherent differences among plants and cultivation environments, geographic authentication and traceability (GAT) of plant origin can be challenging. However, market globalization and improved transportation have increased the value of GAT, particularly for plants from specific, narrow geographic regions to consumers, governments, and manufacturers (Khayatan et al., 2024). To date, the globalization of markets and the enhanced transportation infrastructure have elevated the importance and value of GAT in a narrow region. In this study, we used FT-NIR spectroscopy combined with ML to address this challenge by accurately distinguishing *A. tsaoko* seeds from a distinct origin, Yunnan Province. Our approach achieved identification accuracies of up to 96.97%, successfully classifying samples from origins separated by distances as short as 22.9 km. Such fine-scale discrimination has been predominantly studied in high-value products like olive oil (Wang et al., 2023) and wine (Rodríguez-Bencomo et al., 2011; Styger et al., 2011). Its application to *A. tsaoko* is novel and can serve as a paradigm extendable to other species, thereby advancing quality control and authenticity verification of regional agricultural products.

Previous research utilized combined NIR and ultraviolet-visible light (UV-Vis) spectroscopy to identify *A. tsaoko* fruits originating from five distinct geographical regions within Yunnan province (Liu et al., 2021a). However, this study employed only basic analytic methods, such as principal component analysis (PCA) and partial least squares discriminant analysis (PLS-DA). These methods lack the ability to generalize across different regions and sample types. Furthermore, these studies did not fully address the complex variations in topography, climate, and ecology that exist within Yunnan province. Our study expanded the number of regions sampled to 12. We used a more accurate ML to achieve better geographic authentication.

ML is a program that extracts unknown features from large datasets for prediction or classification. As an analytical technique, it is useful primarily for finding a relationship between inputs and outputs in sample data (Kabir et al., 2021). The results of this study firmly align with the current trajectory in food and agricultural science, where the integration of NIRS with ML is rapidly establishing itself as a versatile and powerful paradigm for non-destructive analysis (Zhang et al., 2022; Miao et al., 2025). This is unequivocally demonstrated by a surge of recent research across diverse applications. For instance, in the context of cereal quality and composition, studies have successfully deployed this combination for rapid protein detection in rice through Raman-NIR fusion (Wang et al., 2023), non-destructive assessment of moisture and fatty acids in rice via hyperspectral imaging (Song et al., 2023), and nutrient quantification in sorghum (Wu et al., 2025). Beyond staple grains, the technique has also proven effective for quality control and defect identification in tubers. Notable examples include the identification of internal defects in potatoes (Semyalo et al., 2024) and the online inspection of blackheart using interpretable deep learning models (Guo et al., 2024). Furthermore, the scope of NIR applications extends to physiological monitoring and origin traceability, with applications ranging from estimating maize leaf water content across growth stages (Ren et al., 2025) to classifying mung beans of different origins (Wu et al., 2023). The convergence of these studies highlights a field moving beyond mere feasibility toward sophisticated, application-specific solutions. This study also provided an in-depth comparison of deep learning architectures. The finding that the CNN model outperformed both the Transformer and a hybrid CNN-Transformer architecture offers valuable insights. It suggests that for the task of geographical authentication of *A. tsaoko* using FT-NIR spectra, the local morphological patterns and short-range dependencies within the spectral data are more discriminative than the long-range, global contextual relationships that Transformers excel at capturing. The underperformance of the more complex hybrid model relative to the standalone CNN can likely be attributed to the limited sample size, which may have hindered the effective training of the increased number of parameters and the learning of meaningful global representations on top of local features. This aligns with the recognized challenge of training deep, complex models on small-scale spectroscopic datasets. Therefore, while hybrid architectures hold theoretical promise, our empirical results indicate that for the present scope, a well-tuned CNN provides a more effective and efficient deep learning solution. Future work, leveraging larger and more diverse multi-seasonal datasets, will be essential to fully unlock the potential of more sophisticated hybrid and ensemble architectures for further improving the robustness and accuracy of geographical traceability models. Our work on *A. tsaoko* traceability thereby contributes to this expanding ecosystem of NIR-AI applications.

It is important to acknowledge the inherent limitations of FT-NIR spectroscopy in pinpointing specific metabolites. The absorption bands in the NIR region arise from broad, overlapping overtones and combination vibrations of fundamental mid-IR modes, primarily involving C–H, O–H, and N–H bonds (Wang et al., 2020). Consequently, while our correlation analysis linked key discriminatory wavenumbers to metabolites quantified by orthogonal methods (GC-MS for volatile compounds), these associations should be interpreted as reflecting general chemical moieties and generating hypotheses, rather than providing definitive identifications of single compounds. The strong correlation between the top SHAP feature (5972 cm^-1^) and key acetate esters, for instance, plausibly indicates the model’s sensitivity to variations in the C-H bonding environment associated with these quality attributes, without implying exclusivity. This perspective reinforces that the observed spectral differences across origins are suggestive of underlying chemical variations that support authentication, while avoiding overinterpretation of the specific biochemical mechanisms.

Beyond the considerations of sample scope, a detailed, class-wise evaluation of our optimal MLP model offers further confidence in its practical utility and reveals insightful nuances (**Supplementary Table 2**). the model achieved perfect classification (Precision, Recall, and F1-score of 1.0) for 10 out of the 12 geographical origins. This high-performance across the vast majority of classes underscores the robustness of the FT-NIR and machine learning approach for fine-scale geographical authentication. The comprehensive performance metrics did, however, identify a specific misclassification between the JP and LS origins. Statistical analysis of the top SHAP-selected spectral features revealed no significant difference between these two classes (**Supplementary Figure 7**), providing a data-driven explanation for this confusion: the chemical profiles of seeds from JP and LS, as captured by the most discriminatory FT-NIR wavelengths, are inherently similar. Crucially, since JP and LS are not geographically adjacent, this similarity is unlikely due to environmental continuum and may instead stem from shared but unknown agricultural practices, soil compositions, or genetic backgrounds. This finding is not a failure of the model but rather a reflection of the authentic, underlying chemical similarity between these two distinct origins. It highlights the sensitivity of our method and suggests that distinguishing these two sites might require incorporating additional, orthogonal data (e.g., genetic markers or elemental analysis). Nevertheless, the model’s performance remains exceptionally high overall, and its ability to clearly delineate origins separated by very short distances (e.g., 22.9 km) is a significant achievement.

This study has certain limitations that should be considered when interpreting the results. The most significant limitation is the sample diversity, as our model was developed and validated using samples collected solely during the 2018 harvest season (36 pooled samples: 12 production regions × 3 mixed pools). This single-season dataset does not account for potential environmental or seasonal variations across different growing seasons, which could impact model generalizability and increases the risk of overfitting. We explicitly acknowledge this limitation. Future studies incorporating samples across multiple seasons and years are essential to validate and further strengthen the robustness of these findings.

Despite this limitation, we indirectly evaluated the reproducibility and transferability of our FT-NIR methodology. We conducted a comparative analysis of our spectral dataset against an independent, publicly available FT-NIR spectral dataset of related Zingiberaceae species (Li et al., 2024). The analysis revealed an exceptionally strong correlation (r > 0.999, **Supplementary Figure 8**). This finding confirms the reproducibility of our spectral acquisition protocol and the consistency of our spectral profiles with established field measurements. Moreover, previous research (Li et al., 2024) indicates that the first overtone of C–H stretching vibration associated with –CH_2_ groups (6000–5400 cm^-1^) serves as a discriminative feature for classifying *A. tsaoko* and *A.* maximum. Consistent with this finding, our analysis identified wavelengths near 5972 and 6000 cm^-1^ as among the most informative features for discrimination. This concordance with previously reported spectral regions further reinforces the robustness of our approach. The reproducibility of these results strengthens confidence in the reliability of our data and suggests the potential for transferring our analytical approach. Nevertheless, future validation with larger and more diverse sample sets of *A. tsaoko* remains an important next step.

## 5 Conclusion

In summary, this study demonstrates that FT-NIR spectroscopy combined with machine learning and SHAP-based feature selection provides a rapid, non-destructive, and highly effective strategy for the precise geographical authentication of *A. tsaoko* seeds. By identifying ten critical spectral features and determining the MLP as the optimal model, we achieved a high identification accuracy of 96.97% for distinguishing seeds from 12 narrowly separated geographical origins. While the model’s performance is promising, its generalizability is currently limited by the single-harvest dataset. Nevertheless, this work establishes a powerful and practical framework, implemented via a web application, for the quality control and origin traceability of *A. tsaoko* and potentially other high-value agricultural products.

## Data Availability

The datasets presented in this study can be found in online repositories. The names of the repository/repositories and accession number(s) can be found in the article/**Supplementary Material**.

## Supplementary material

Supplementary Figure 1 Morphological characteristics of *Amomum tsaoko* from 12 different geographical origins. For each pair, the left panel shows the powdered form, and the right panel shows a representative individual fruit.

Supplementary Figure 2 Architecture of CNN

Supplementary Figure 3 Architecture of Transformer

Supplementary Figure 4 Disutribution comparison between orgin data and SMOTE data.

Supplementary Figure 5 Disutribution comparison between orgin data and bootstrap oversampling data.

Supplementary Figure 6 Disutribution comparison between orgin data and random oversampling data.

Supplementary Figure 7 Statistical analysis of the top SHAP-selected spectral features between the JP and LS origins.

Supplementary Figure 8 High consistency between FT-NIR spectra from the current study and published data (Li et al., 2024).

Supplementary Table 3 Raw spectral data.
