# Supplementary figures and images for: Geographic authentication of Amomum tsaoko seeds using fourier transform-near infrared spectroscopy combined with machine learning techniques and feature reduction analysis
Source: Front Plant Sci. 2026 Jan 22;16:1717851. doi: 10.3389/fpls.2025.1717851 (PMC12872912; doi:10.3389/fpls.2025.1717851)

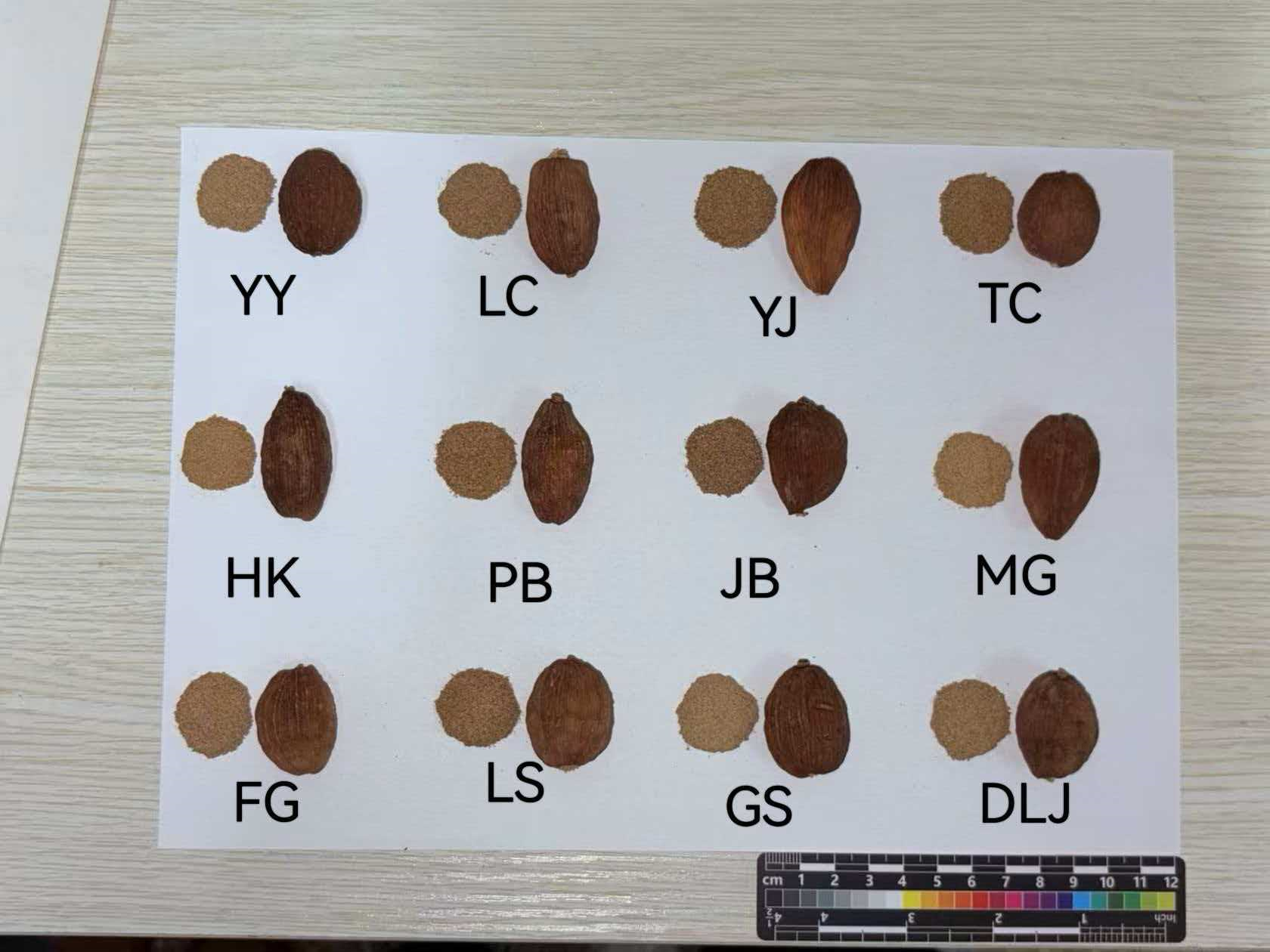

Supplement: Supplementary Figure 1 — Morphological characteristics of Amomum tsaoko from 12 different geographical origins. For each pair, the left panel shows the powdered form, and the right panel shows a representative individual fruit. [file Image1.tiff]

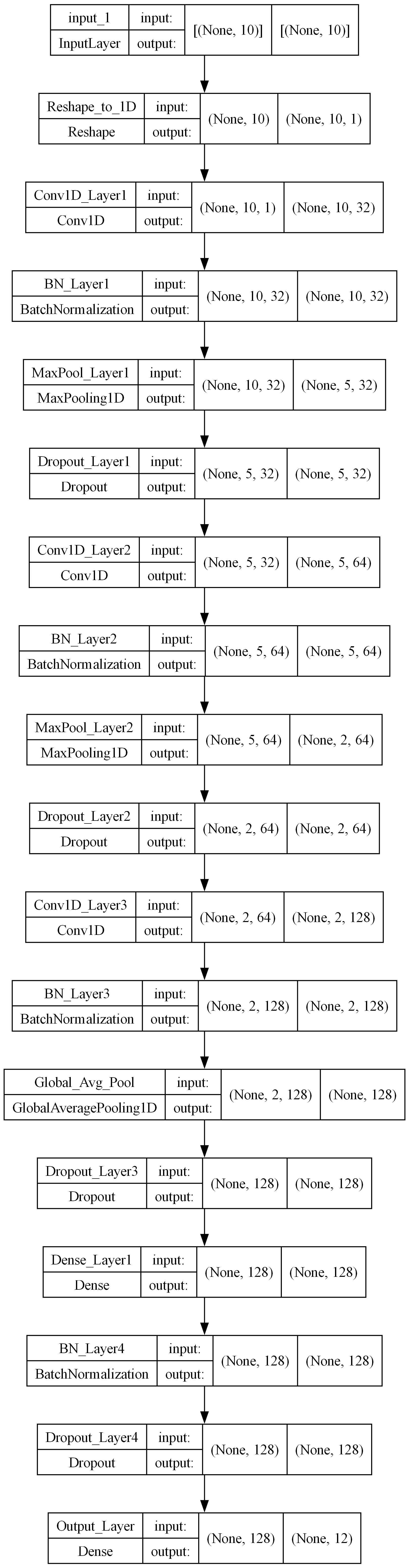

Supplement: Supplementary Figure 2 — Architecture of CNN [file Image2.tiff]

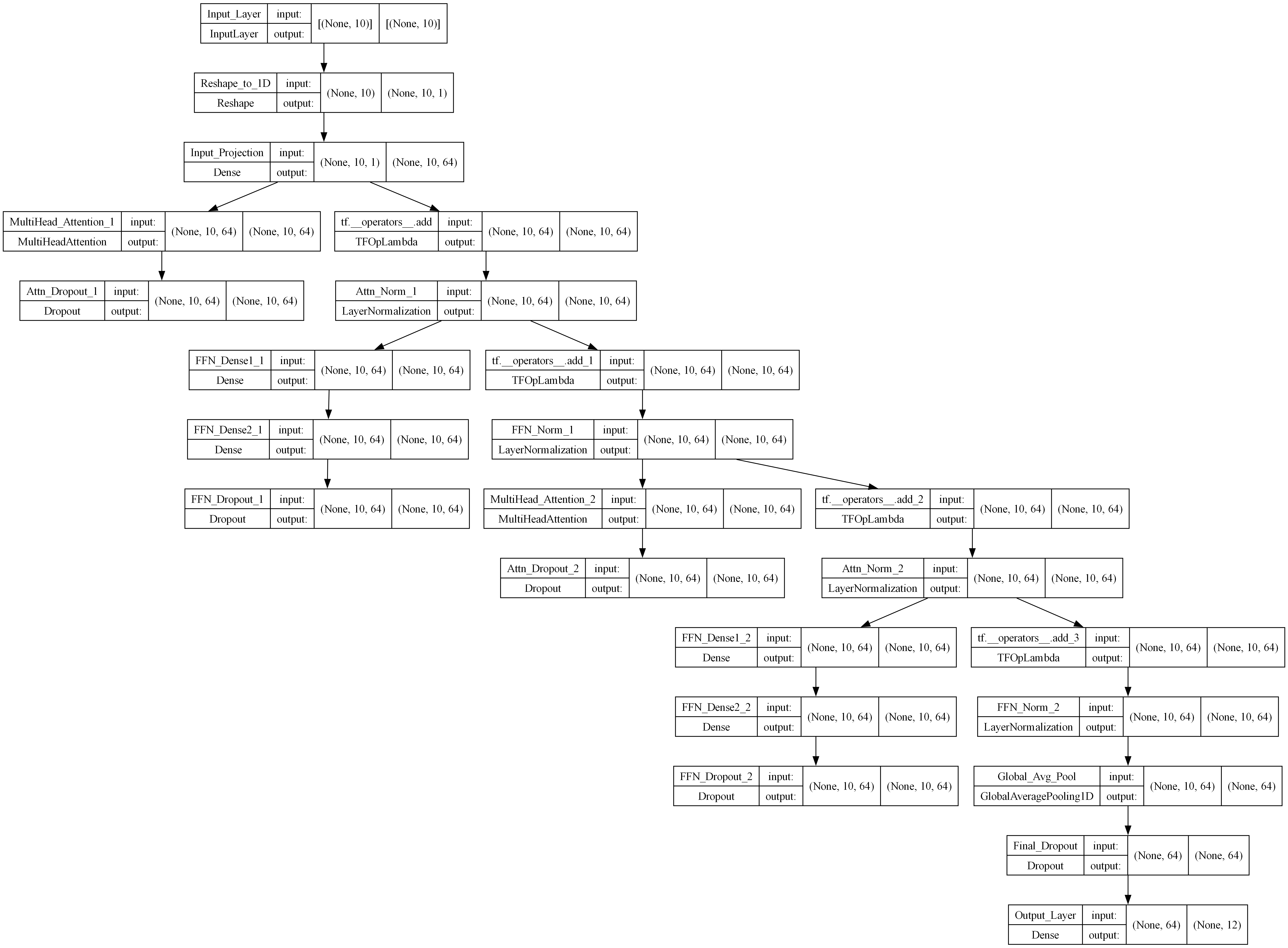

Supplement: Supplementary Figure 3 — Architecture of Transformer [file Image3.tiff]

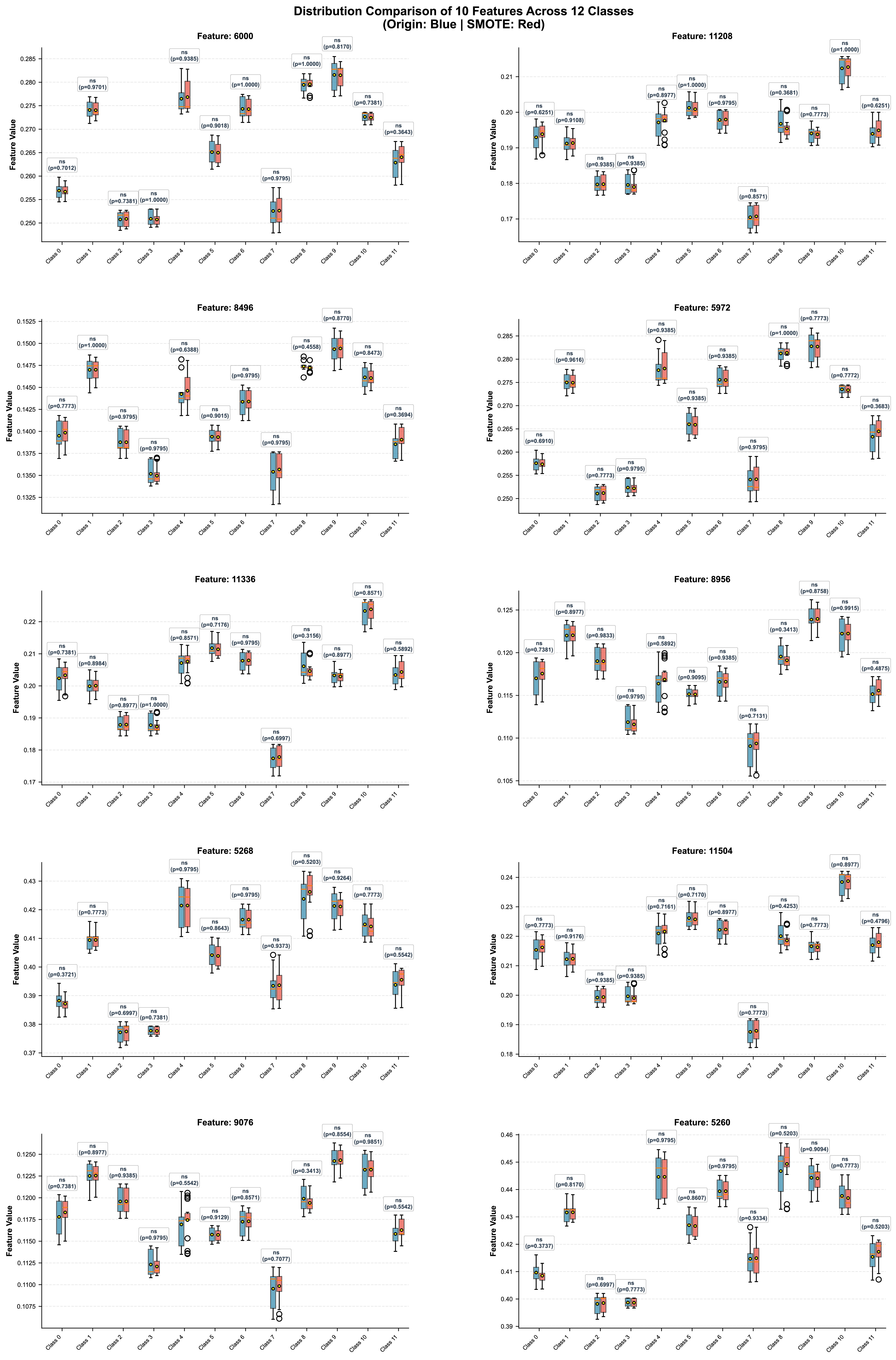

Supplement: Supplementary Figure 4 — Disutribution comparison between orgin data and SMOTE data. [file Image4.tiff]

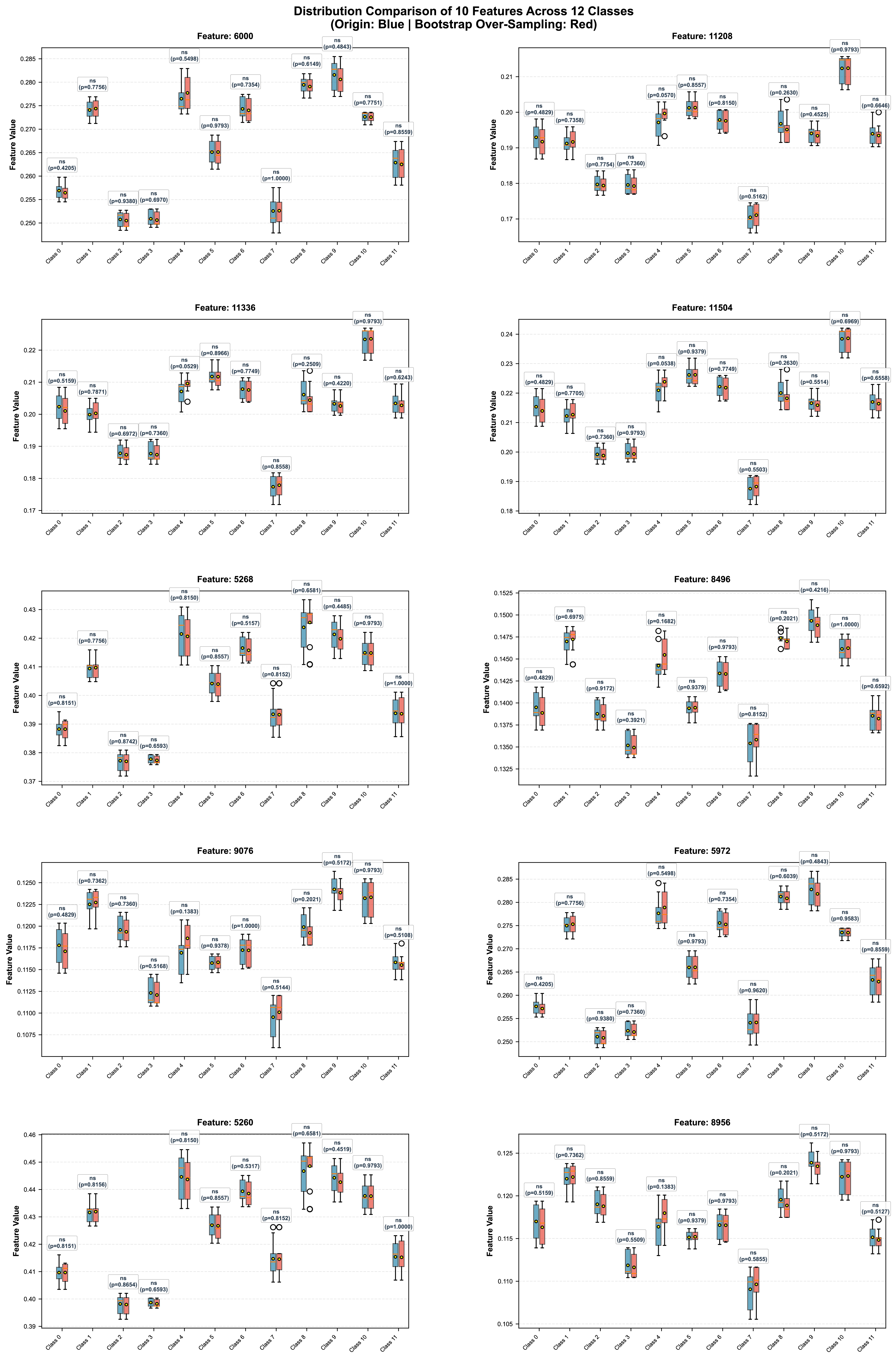

Supplement: Supplementary Figure 5 — Disutribution comparison between orgin data and bootstrap oversampling data. [file Image5.tiff]

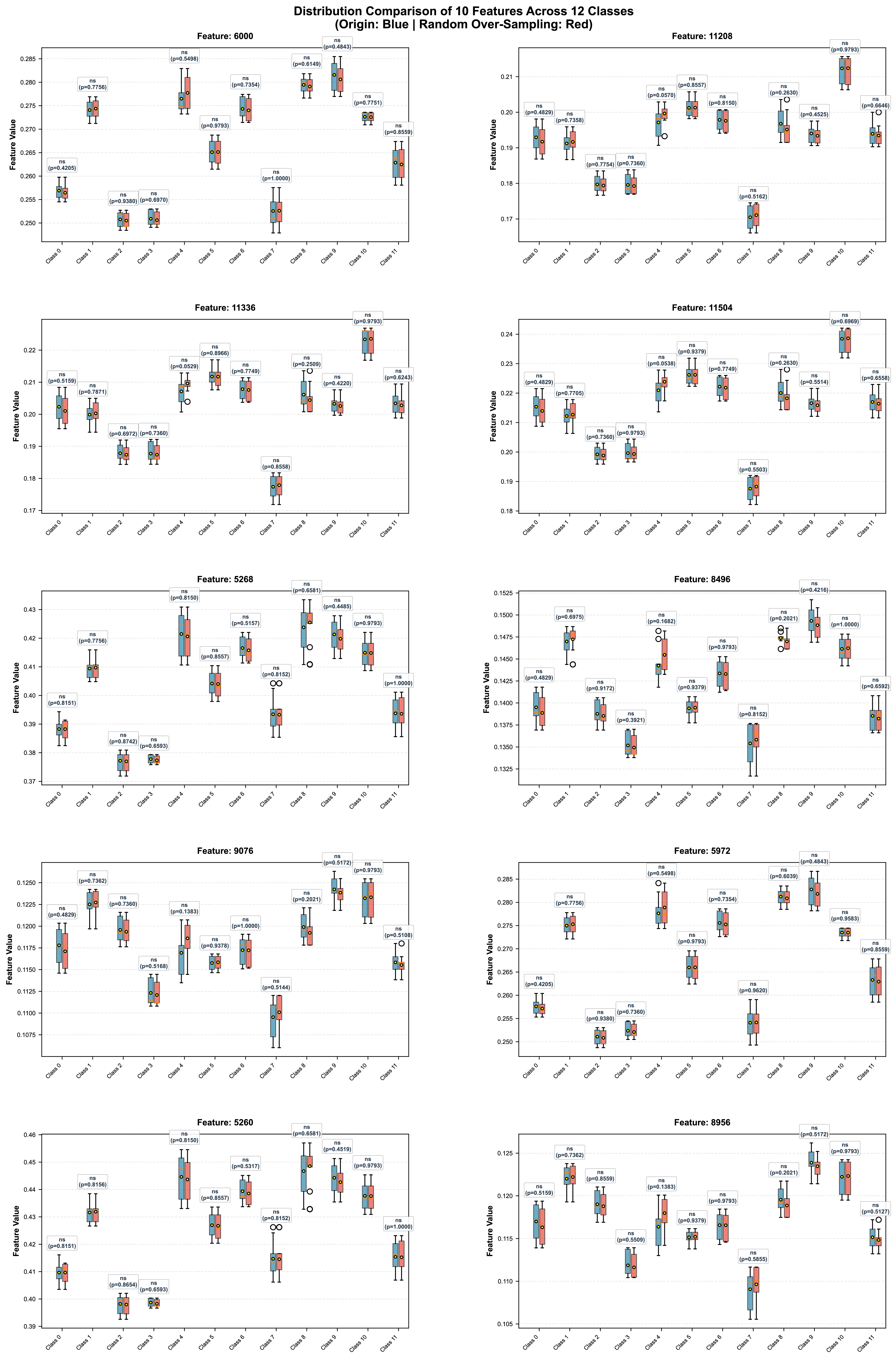

Supplement: Supplementary Figure 6 — Disutribution comparison between orgin data and random oversampling data. [file Image6.tiff]

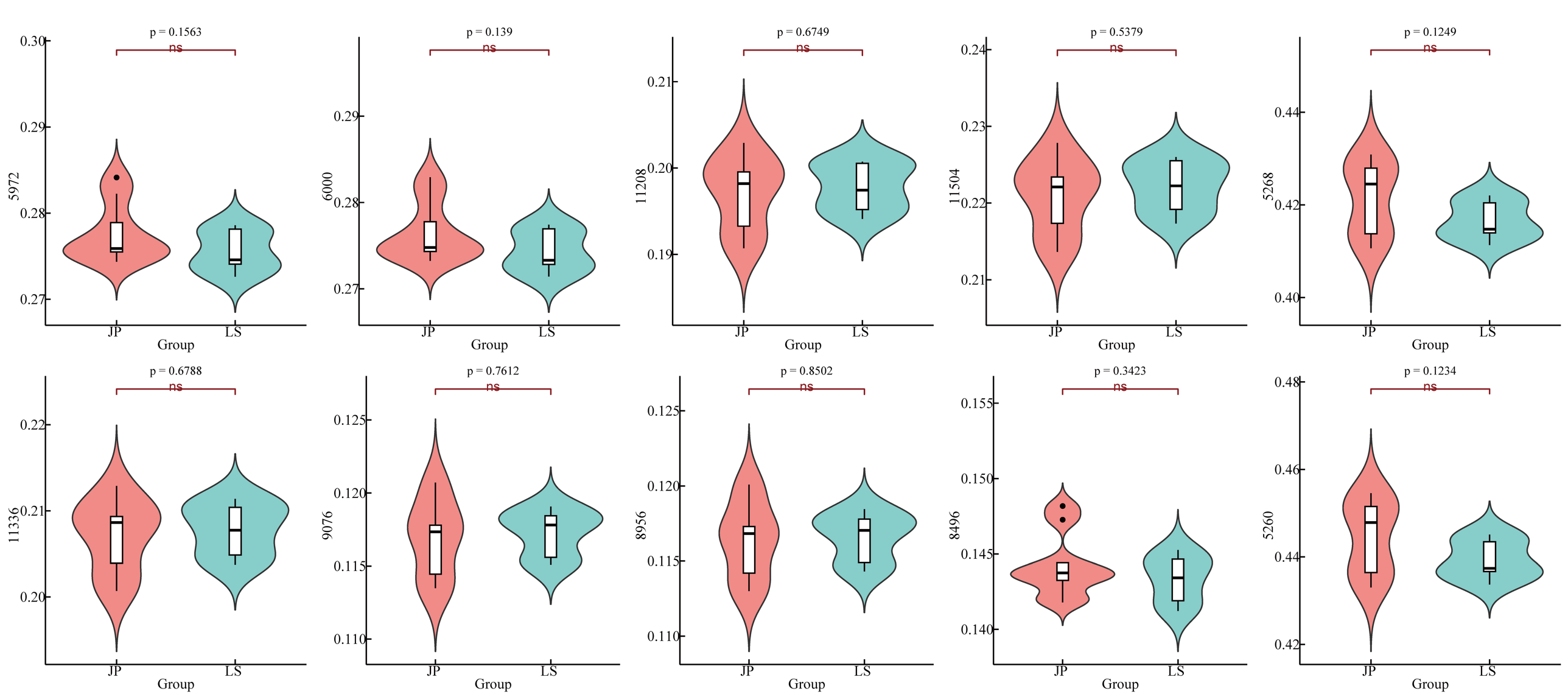

Supplement: Supplementary Figure 7 — Statistical analysis of the top SHAP-selected spectral features between the JP and LS origins. [file Image7.tiff]

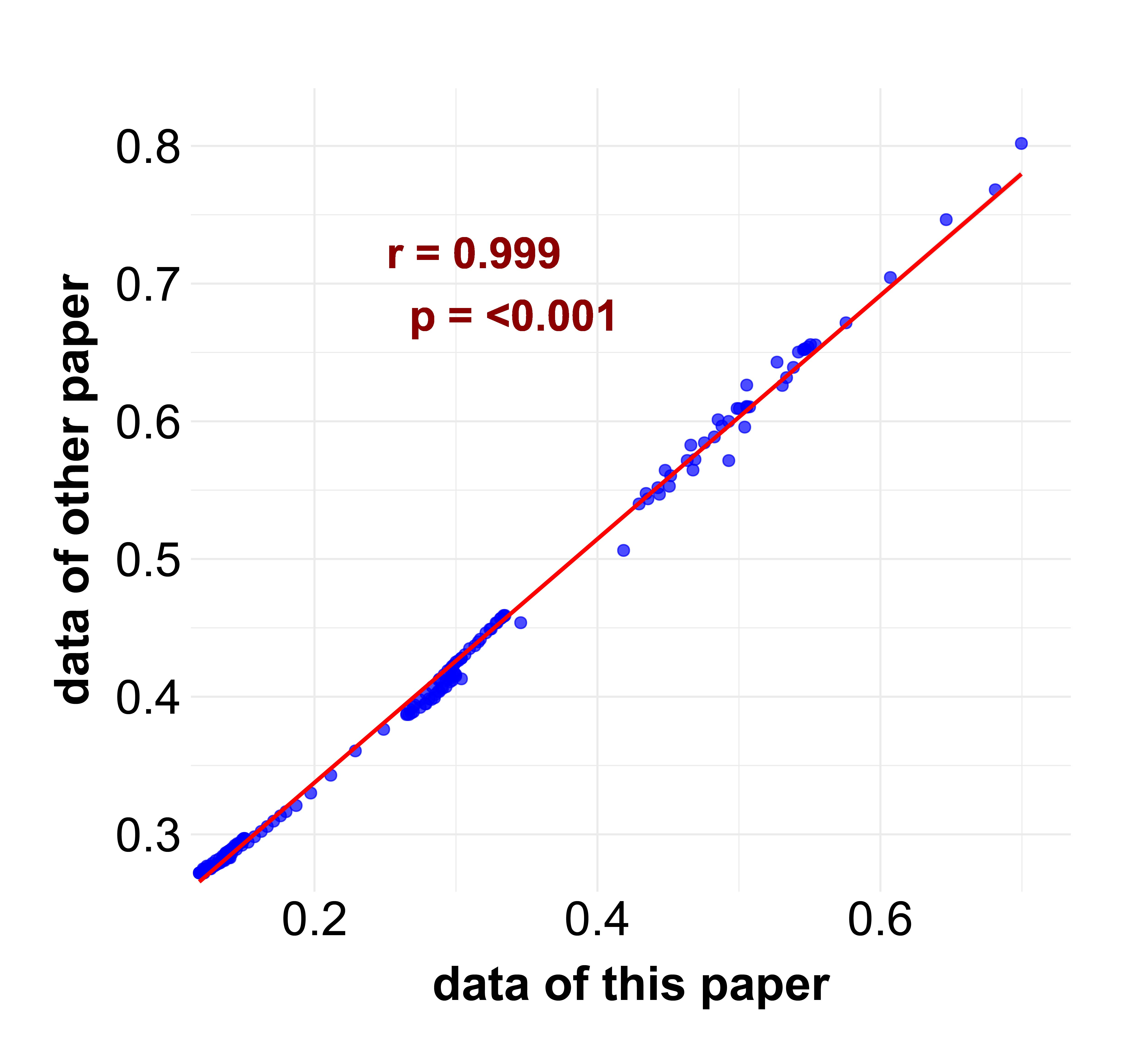

Supplement: Supplementary Figure 8 — High consistency between FT-NIR spectra from the current study and published data (Li et al., 2024). [file Image8.tiff]
