# Supplementary material for: Geographic authentication of Amomum tsaoko seeds using fourier transform-near infrared spectroscopy combined with machine learning techniques and feature reduction analysis
Source: Front Plant Sci. 2026 Jan 22;16:1717851. doi: 10.3389/fpls.2025.1717851 (PMC12872912; doi:10.3389/fpls.2025.1717851)
Supplement: Supplementary file 10 [file Table1.docx]

**Supplementary Table S1. A comparative overview of analytical methods for the authentication and analysis of *Amomum tsaoko***

| Analytical Technique | Discrimination Target / Focus | Sample Scale/ Origin Number | Key Findings/ Application | Major Limitations | Reference |
| --- | --- | --- | --- | --- | --- |
| **GC-MS & NIR** | Species: *A. tsaoko* vs. *A. paratsao-ko* | Not Specified / 2 Species | Identified differential metabolites and used NIR for species classification. | Focused on species-level discrimination, not fine-scale geographical origin. | [13] |
| **EST-SSR Markers** | Genetic diversity and population structure | 9 Populations | Revealed high genetic diversity and classified populations into distinct groups. | A molecular biology technique requiring DNA extraction, not directly applicable for rapid, non-destructive quality control of commercial products. | [14] |
| **NIR & UV-Vis Spectroscopy** | Geographical origin of fruits | 5 Regions in Yunnan | Combined multi-platform spectra with PCA and PLS-DA for geographical traceability. | Limited to a coarse geographical scale (5 regions); relied on basic chemometric methods (PCA, PLS-DA) with lower resolution. | [15] |
| **Multi-element Fingerprinting (ICP-MS)** | Geographical origin of seeds | 12 Populations in Yunnan | Achieved high accuracy in authenticating seeds from 12 narrowly defined geographical origins. | Destructive method; requires complex sample preparation and sophisticated instrumentation; lower throughput and higher cost. | [16] |
| **FT-NIR & Machine Learning (Current Study)** | **Fine-scale geographical origin of seeds** | **12 narrowly separated Populations in Yunnan** | **Achieved 96.97% accuracy using MLP; Identified top 10 discriminatory features via SHAP; Successful discrimination over short distances** | **Model based on single-season data; Generalizability to other seasons requires further validation.** | **This Work** |
