# Supplementary material for: Geographic authentication of Amomum tsaoko seeds using fourier transform-near infrared spectroscopy combined with machine learning techniques and feature reduction analysis
Source: Front Plant Sci. 2026 Jan 22;16:1717851. doi: 10.3389/fpls.2025.1717851 (PMC12872912; doi:10.3389/fpls.2025.1717851)
Supplement: Supplementary file 11 [file Table2.docx]

**Supplementary Table S2. Precision, Recall, and F1-score for each of the 12 geographical origins (classes) based on the confusion matrix of our optimal MLP model (7:3 split)**

| **Class** | **TP** | **FP** | **FN** | **Precision** | **Recall** | **F1_Score** |
| --- | --- | --- | --- | --- | --- | --- |
| **0** | **2** | **0** | **0** | **1** | **1** | **1** |
| **1** | **3** | **0** | **0** | **1** | **1** | **1** |
| **2** | **3** | **0** | **0** | **1** | **1** | **1** |
| **3** | **3** | **0** | **0** | **1** | **1** | **1** |
| **4** | **2** | **0** | **1** | **1** | **0.6667** | **0.8** |
| **5** | **3** | **0** | **0** | **1** | **1** | **1** |
| **6** | **3** | **1** | **0** | **0.75** | **1** | **0.8571** |
| **7** | **3** | **0** | **0** | **1** | **1** | **1** |
| **8** | **3** | **0** | **0** | **1** | **1** | **1** |
| **9** | **2** | **0** | **0** | **1** | **1** | **1** |
| **10** | **2** | **0** | **0** | **1** | **1** | **1** |
| **11** | **3** | **0** | **0** | **1** | **1** | **1** |
